# Selection Instability and External Performance of Transcriptomic Models Beyond Age, Sex and Stage in Resected Lung Adenocarcinoma

**DOI:** 10.3390/cancers18203361

**Published:** 2026-10-17

**Authors:** Zhihao Lei, Chenxi Zou, Wei Zhao

**Affiliations:** 1 Department of Statistics and Data Science, Cornell University, Ithaca, NY 14853, USA; 2 Department of Pulmonary and Critical Care Medicine, The First Medical Center, Chinese PLA General Hospital, Beijing 100853, China; 3 Senior Department of Pulmonary and Critical Care Medicine, The Eighth Medical Center of Chinese PLA General Hospital, Beijing 100091, China

**Keywords:** lung adenocarcinoma, machine learning, external validation, survival analysis, calibration, transcriptomics, transportability

## Abstract

Background/Objectives: We evaluated molecular selection stability and external prediction beyond age, sex and broad pathological stage in resected stage I–II lung adenocarcinoma. Methods: Models were developed in 228 patients from two sources and frozen before evaluation in four historical cohorts containing 778 patients. The primary comparison added pathway scores using elastic-net Cox regression. Secondary analyses examined a gene model, 200 development bootstrap resamples, alternative modeling specifications and locally updated baselines. Prediction error was measured by the integrated Brier score over 1–3 years (IBS); negative molecular-minus-clinical differences (ΔIBS) favored molecular models. Evaluation intervals used 2000 paired bootstrap resamples. Results: The pathway model retained no molecular terms. The final gene model retained 16 genes, whereas both source-held-out fits and 159/200 development resamples retained none. Penalties of 0.5, 0.15 and 0.05 were selected in 160, 38 and 2 resamples. Alternative specifications changed gene retention from 0 to 61 and pathway retention from 0 to 5. Frozen gene-model ΔIBS ranged from −0.0034 to 0.0055 across cohorts. Its equal cohort mean was 0.0016 (95% CI, −0.0012 to 0.0042), compared with −0.0008 (−0.0024 to 0.0007) after cross-fitted local baseline updating. The clinical model predicted a 3-year mortality of 19.6% in Japan and 22.6% in Moffitt, versus observed values of 9.1% and 28.6%. Conclusions: Molecular selection was unstable, and incremental performance depended on specification and population. The results characterize these learning procedures and their limited development sample; broader transcriptomic prognostic value and contemporary clinical utility remain unestablished.

## Simple Summary

A prognostic model must learn a stable signal and give reliable predictions in patients from varying populations. We developed transcriptomic models using 228 patients with resected lung adenocarcinoma and evaluated them in four historical cohorts containing 778 patients. The reference model used age, sex and broad pathological stage. The main pathway model retained no molecular terms. A gene model retained 16 genes, but 159 of 200 repetitions of model development selected none. Alternative modeling choices changed molecular retention without producing lower prediction error in every cohort. Predicted mortality also differed from observed mortality, particularly in Japan and Moffitt. Updating baseline risk using separate local patients changed the model comparisons. These findings identify selection instability and population-specific calibration as practical constraints on the evaluated prediction procedures. They do not establish the absence of prognostic information in transcriptomic measurements.

## 1. Introduction

Survival after lung adenocarcinoma resection varies within pathological stage groups. Gene expression measurements can capture tumor characteristics associated with that variation. The Director’s Challenge established the importance of the multisite evaluation of expression-based prognostic models [1], and the prospective validation of the ORACLE clonal expression biomarker demonstrated a route from biological development to a predefined prognostic test [2]. These studies motivate assessing both the information learned from transcriptomics and the performance of its transferred prediction rule.

Public-data studies have developed diverse lung cancer signatures, including immunosenescence models and essential gene expression subtypes [3,4]. Interpretable machine learning has also been applied to brain-metastasis prediction in breast cancer [5], a different endpoint and population. For postoperative survival, the unresolved question is whether a molecular learning procedure adds predictive information when its preprocessing, coefficients and baseline survival are transferred together. Age, sex and broad pathological stage provide a harmonizable benchmark for this comparison across historical lung cancer cohorts.

Discrimination, calibration and selection stability address different requirements of a prognostic model. Ranking can remain informative while absolute risks are systematically inaccurate [6,7]. Transparent development and external evaluation are therefore essential [8]. In small development samples, penalization does not eliminate uncertainty in tuning or predictor selection [9,10,11], and high-dimensional cancer signatures can depend strongly on the sampled patients [12]. Evaluating one frozen model and repeating the learning procedure address complementary parts of this uncertainty.

We assessed clinical–transcriptomic learning procedures in resected stage I–II lung adenocarcinoma. Four external cohorts remained outside parameter fitting and model selection. We examined molecular selection stability, cohort-specific prediction error, discrimination and calibration and compared complete frozen models with versions whose baseline hazards were updated using separate local patients.

## 2. Materials and Methods

### 2.1. Study Design and Analysis Governance

This retrospective study used publicly available, deidentified data. An internal analysis specification recorded on 15 September 2026 defined eligibility, cohort roles, feature coverage, candidate models, tuning grids and the primary contrast before model fitting and the inspection of external predictive performance. Its archived contents and provenance are reproduced in Supplementary File S1. This was an internally documented plan, without prospective registration or an independently certified timestamp. All six original final models, including preprocessing parameters and baseline survival functions, were frozen before external predictions were generated.

The primary estimand concerned the transfer of complete models to four specified external populations. The principal molecular comparison used pathway scores; gene-level and other algorithmic comparisons were secondary. Development resampling, complementary aggregation, censoring diagnostics and locally updated baselines provide supporting evidence with their original exploratory status. Alternative regularization, variance filters and pathway dimension reduction were post hoc specification sensitivities. These analyses did not replace the original frozen models or select a preferred model using external outcomes.

### 2.2. Cohort Selection and Source Separation

Complete processed expression matrices, platform annotations and sample metadata were obtained from the Gene Expression Omnibus (GEO) for GSE68465, GSE72094, GSE42127, GSE50081 and GSE31210. The corresponding studies described the Director’s Challenge, Moffitt, MD Anderson, Toronto and Japanese early-stage cohorts [1,13,14,15,16]. Download integrity was verified before analysis.

Eligible patients were adults with adenocarcinoma, reported pathological stage I or II, known sex, and positive overall survival follow-up with an interpretable event indicator. Normal lung, other histologies, missing or out-of-range stages and invalid survival observations were excluded. In GSE68465, historical pathological TN combinations were mapped as N0T1–2 to stage I and N1T1–2 or N0T3 to stage II. Other cohorts retained their reported stage groups. Unavailable tumor measurements precluded reassignment to a common contemporary TNM edition.

Development used the Michigan and Memorial Sloan Kettering sources of GSE68465. Its H. Lee Moffitt and pooled Dana–Farber/Canadian sources were excluded to reduce potential institutional overlap with GSE72094 and GSE50081. Evaluation cohorts did not contribute to fitting imputers, standardizers, coefficients, baseline survival or hyperparameters. Within-cohort identifiers and expression profiles were screened for exact duplication. Cross-cohort rank-profile similarity provided an additional overlap screen, although historical patient identities could not be linked conclusively.

Overall survival denoted death from any cause, with living patients censored at final follow-up. Surgical time origins were supported by source descriptions and published cohort analyses [14,17,18]. Follow-up was converted from months to years for GSE68465 and GSE42127 and from days to years for GSE72094 and GSE31210; GSE50081 already reported years. Patient-level surgical dates were unavailable. TCGA was excluded because the commonly used clinical resource has a diagnosis-based time origin [19]. Endpoint fields and source locations appear in Table S1.

Resection completeness and treatment were not uniformly recorded. The population comprises reported surgical cohorts with heterogeneous postoperative management. One development patient had a positive-margin flag. A sensitivity analysis excluded 22 GSE31210 patients flagged for incomplete resection or adjuvant therapy. The development sample was determined by eligible records in the two institutionally separated sources, rather than an a priori sample size calculation. Cox fitting used all 96 observed development deaths; 46 occurred by the 3-year evaluation horizon. This provides limited information for 43 candidate pathways or 500 candidate genes in addition to three clinical variables. Formal prediction-model sample size planning depends on the anticipated signal, candidate complexity and acceptable shrinkage [20,21]; the available sample was not assumed to meet such a target.

### 2.3. Gene Mapping and Pathway Representation

Probe annotations from GPL96 (Affymetrix Human Genome U133A Array), GPL15048 (Rosetta/Merck Human RSTA Custom Affymetrix 2.0 Microarray), GPL6884 (Illumina HumanWG-6 v3.0 Expression BeadChip), and GPL570 (Affymetrix Human Genome U133 Plus 2.0 Array) were mapped to gene symbols. The platforms were manufactured by Affymetrix, Inc. (Santa Clara, CA, USA) or Illumina, Inc. (San Diego, CA, USA). Probes with absent or ambiguous multi-gene symbols were removed; multiple probes per gene were summarized by their median processed expression within a sample. The predictor universe was the intersection across all four platforms. This was an outcome-independent, evaluation-platform-informed design: evaluation assay annotations contributed to feature eligibility, while patient outcomes and expression distributions did not. Consequently, the design evaluates transport across these known compatible platforms rather than an unknown future assay.

Genes in the fixed universe were ranked in ascending order within each patient, with average ranks for ties. Pathway scores followed a centered, normalized mean-rank formulation [22]: for k measured pathway genes among N background genes, score = [mean(rank) − (N + 1)/2]/(N − k). Hallmark gene sets came from MSigDB release 2025.1.Hs [23]. Pathways required at least 10 measured genes and 80% coverage of original membership. Gene aliases were not substituted to increase coverage.

Downstream ranks and scores were independent of the accompanying patient batch. The input expression measurements nevertheless retained study-specific array processing performed by the original investigators. No joint development–evaluation batch correction, external standardization or outcome-based probe selection was applied.

### 2.4. Models and Development Procedure

The clinical reference model included age, recorded sex and broad pathological stage group. Continuous age was modeled linearly; sex and stage II were binary variables. All predictors were median-imputed and standardized using the relevant training fold. No primary clinical variable was missing among eligible patients. The reference Cox model used Breslow ties and a small ridge penalty of 0.01.

The primary molecular model added eligible Hallmark scores using Cox elastic-net regression [24,25]. Clinical coefficients were unpenalized; molecular coefficients used an L1 mixing fraction of 0.5 and penalties of 0.005, 0.015, 0.05, 0.15 or 0.5. Equal L1 and L2 mixing balanced sparse selection with the shrinkage of correlated predictors, while the five-value grid limited the original tuning search. The gene comparator retained the 500 genes with the highest within-training-fold rank variance to reduce dimensionality without outcome-based screening. These were pragmatic specifications rather than established optimal settings. Random survival forest and Cox-loss boosting used compact prespecified grids (Table S4); conclusions about these algorithms concern those implementations.

An additional comparator refitted the five immunosenescence risk model (ISRM) genes IL7, CD40LG, CX3CR1, TLR3 and TLR2 with the same rank-based elastic-net pipeline and clinical covariates [3]. This comparison refitted the published gene set using this study’s rank units and baseline estimation; it evaluated a different prediction rule from the published score. The prior use of these public cohorts by signature studies limits historical independence.

Final hyperparameters minimized the equal-source mean IBS in leave-one-development-source-out evaluation. Internal assessment used an outer source-held-out procedure with three-fold event-stratified tuning within the remaining source. Thus, the final and nested fits used different training samples and tuning partitions. Every imputer, standardizer and variance filter was refitted within training data. Failed or nonconvergent candidates received an infeasible tuning score. Chosen settings were then fitted to all development patients, and preprocessing, coefficients and baseline survival were frozen together. Source-separated evaluation addresses between-source transfer [26], but only two sources were available, and no external outcome informed final-model selection.

### 2.5. Performance Measures and Uncertainty

The primary contrast was pathway-minus-clinical integrated Brier score (ΔIBS) over 1–3 years; negative values favor the molecular model. The Brier score was evaluated at 25 equally spaced times and integrated by the trapezoidal rule. Marginal inverse probability of censoring weights (IPCW) was estimated separately within each evaluated cohort. Deaths at T ≤ t received weight 1/G(T−), and patients observed beyond t received weight 1/G(t), where G denotes censoring survival [27,28]. Earlier censored observations contributed zero. This estimator assumes that censoring is independent of survival within the evaluated cohort. The primary censoring Kaplan–Meier calculation retained deaths tied with censoring in its denominator; an event-first convention was assessed separately.

Cohort-specific contrasts were the basis for inference about transport. The prespecified equal cohort summary, ΣΔIBS/4, describes the mean contrast across these four specific populations, each weighted 25%. It is neither a patient-level average nor an estimate over a random population of institutions. Complementary summaries used sample size weights n/778 and the within-cohort relative contrast 100 × ΔIBS/IBSclinical. The latter expresses error change relative to the benchmark within each population; it is not the proportion of all explainable prediction error. Descriptive heterogeneity used Cochran’s Q and I^2^, with within-cohort variances estimated by bootstrap.

For frozen-model evaluation, 2000 paired patient bootstrap resamples per cohort recomputed censoring weights. Moreover, 95% confidence intervals (CIs) were calculated, and independent cohort draws were combined using the stated summary weights. These intervals condition on the fitted models and the selected cohorts. Development uncertainty was examined separately by resampling the learning procedure.

Secondary measures included Uno’s concordance statistic truncated at 3 years [27] and cumulative/dynamic AUC at 3 years [29]. At 1, 2 and 3 years, calibration compared mean predicted mortality with Kaplan–Meier mortality and its CI. Horizon-specific slopes used a Cox model with frozen log cumulative predicted hazard as the predictor and follow-up truncated at that horizon [7]. Three-year grouped displays used development-derived risk tertiles. No external cutpoint was optimized.

The technical support criteria for 3-year evaluation were G(3) ≥ 0.20 and at least 30 patients still under observation. These criteria establish computability rather than stable late-horizon estimation. Secondary contrasts were exploratory, and an interval containing zero was not interpreted as equivalence.

### 2.6. Sensitivity and Recalibration Analyses

Molecular selection was assessed in both nested source-held-out fits and 200 source-stratified development bootstrap resamples. Each resample repeated filtering, source-based penalty selection, preprocessing and final fitting. Copies of a patient remained in the same source. We recorded penalty frequencies, retained gene counts, gene reselection and overlap with the final 16-gene set. Spearman correlations compared resampled and frozen total and molecular-only prognostic indices in each original cohort. Molecular correlations were undefined for collapsed fits. These diagnostics characterize learning-procedure variability separately from the conditional external intervals [9,11].

For censoring sensitivity, the frozen clinical and gene models were compared over 1–2, 1–2.5 and 1–3 years. IPCW distributions and effective sample size, (Σw)^2^/Σw^2^, were evaluated at 1, 2, 2.5 and 3 years. Effective sample size describes the weighted outcome contributions and differs from the number still at risk. Additional 1–3-year estimates capped weights at 3, 5 or 10. Each configuration used 2000 paired bootstrap resamples with censoring weights recomputed.

Baseline updating evaluated a local adaptation procedure for the frozen linear predictors. Identical five-fold event-stratified splits were used for the clinical and gene models within each external cohort. Coefficients remained fixed; each baseline hazard was estimated using Breslow increments in four folds and applied to the held-out fold. Paired ΔIBS compared the two locally updated models. In 2000 bootstrap replicates, patients were resampled within original folds, both baseline hazards were re-estimated and censoring weights were recomputed. All copies of a patient remained in one fold. These intervals include local baseline-estimation variability conditional on the original coefficients and fold allocation. This procedure uses local outcomes and evaluates a different deployment setting from complete-model transport.

Proportional-hazards diagnostics tested interactions with centered log event time using global efficient score tests with Breslow ties and administrative truncation at 3 years. Tests were applied to each frozen prognostic index and jointly to age, sex and stage, with the frozen molecular index added for the gene model. Local nuisance coefficients were estimated for the diagnostic null model; prediction coefficients remained unchanged. Such time-interaction diagnostics address departures from constant hazard ratios [30]. Age functional form was assessed by adding one nonlinear restricted-cubic-spline term with development-derived knots at the 10th, 50th and 90th percentiles and a one-degree-of-freedom likelihood-ratio test. Benjamini–Hochberg adjustment was applied separately to the 20 proportional-hazards and 10 age tests.

Other exploratory comparisons examined stage I patients, original prognosis-eligible GSE31210 patients, smoking-extended models where available, and clinical-only forest and boosting controls using the same algorithmic grids and development-source tuning. A downstream batch-composition diagnostic evaluated fixed-patient scores across 100 sampled batches at each of three batch sizes.

Post hoc specification sensitivity varied the elastic-net mixing fraction (0.1 or 0.9), the gene-variance filter (100 or 1000 candidates) and the penalty grid (13 values spanning 0.005–0.5). An alternative pathway representation used five principal components fitted within each training fold, with ridge-penalized component coefficients and unpenalized clinical terms. Each specification selected its penalty using development sources only and was frozen before its external predictions were computed. All configurations were evaluated against the same original clinical model using paired 2000-resample intervals; these post hoc intervals were unadjusted for multiplicity (Supplementary Methods S8).

### 2.7. Software and Ethics

Analyses used Python 3.12, scikit-survival 0.28.0 [31], scikit-learn 1.9.1, lifelines 0.30.3, NumPy2.5.3, pandas 3.0.5 and SciPy 1.18.1, with random seed 20260915. Matplotlib 3.11.2 figures were exported as 600-dpi TIFFs. Computational checks covered survival-probability bounds and monotonicity, disjoint development/evaluation identifiers, prediction reproducibility after model freezing, censoring-estimator agreement with an independent Kaplan–Meier calculation, and the agreement of the uncensored Brier score with ordinary squared error.

This study involved no new recruitment, identifiable records, specimen collection, animal procedures or laboratory experiments. According to the authors’ determination, additional ethical approval and participant consent were not required for this public, deidentified secondary analysis. No institutional exemption number was assigned.

## 3. Results

### 3.1. Cohort Composition and Measurement Coverage

**Figure 1:**
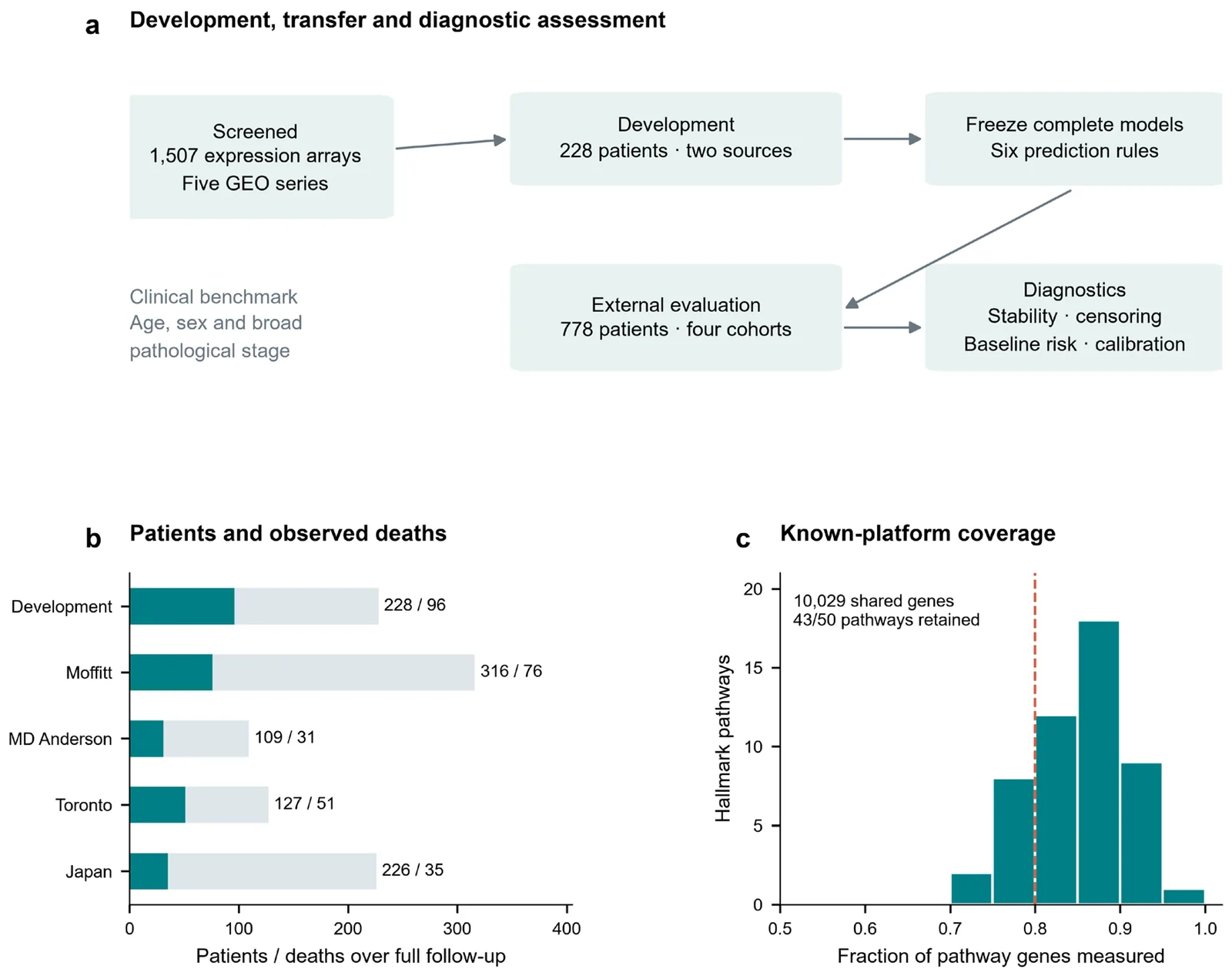
Cohort assembly and feature coverage. (**a**) Complete prediction models were developed in two sources and frozen before evaluation in four external cohorts. Screened records are deposited expression arrays rather than verified unique patients across all deposits. Diagnostic analyses characterize the learning and transfer procedure. (**b**) Light bars show eligible patients; teal bars show deaths over all available follow-up. (**c**) The outcome-independent, evaluation-platform-informed gene intersection supported 43 of 50 Hallmark pathways; the dashed line denotes 80% coverage. GEO, Gene Expression Omnibus.

Among 1507 deposited arrays, 1006 patients met the eligibility and source-separation criteria: 228 for development and 778 for external evaluation (Figure 1; Table 1). Development comprised 145 Michigan and 83 Memorial Sloan Kettering patients, with 96 deaths overall and 46 by 3 years. Moffitt, MD Anderson, Toronto and Japan contributed 316, 109, 127 and 226 patients, with 76, 31, 51 and 35 deaths, respectively. No exact duplicate identifiers or expression profiles were detected within eligible cohorts.

The platforms shared 10,029 mapped genes, supporting 43 of 50 Hallmark pathways under the 80% coverage rule. E2F targets (78.5%) and MYC targets V2 (77.6%) fell below the threshold, while G2M checkpoint and MYC targets V1 were retained (Table S3). The clinical variables were complete, but smoking and treatment information differed in availability across cohorts.

### 3.2. Molecular Selection Depended on the Development Sample

The original pathway and immunosenescence models selected penalty 0.15 and retained no molecular coefficients. Their predictions reduced to clinical components; near-zero contrasts with the clinical reference reflect clinical-penalty and numerical differences, rather than an estimate of molecular benefit.

The full-development gene model retained 16 of 500 candidates (Table 2). Penalty 0.15 achieved mean source-held-out IBS 0.115166, compared with 0.115645 at penalty 0.5, a difference of 0.000479. Both nested fits instead selected 0.5 and retained no molecular terms. Their training samples contained 83 patients/23 deaths and 145 patients/73 deaths, with tuning performed within the remaining source (Tables S5 and S27). The final 16-gene model is the prespecified learning procedure’s output on all development patients, retained as the fixed object of external evaluation; its selection does not establish a stable gene signature.

**Figure 2:**
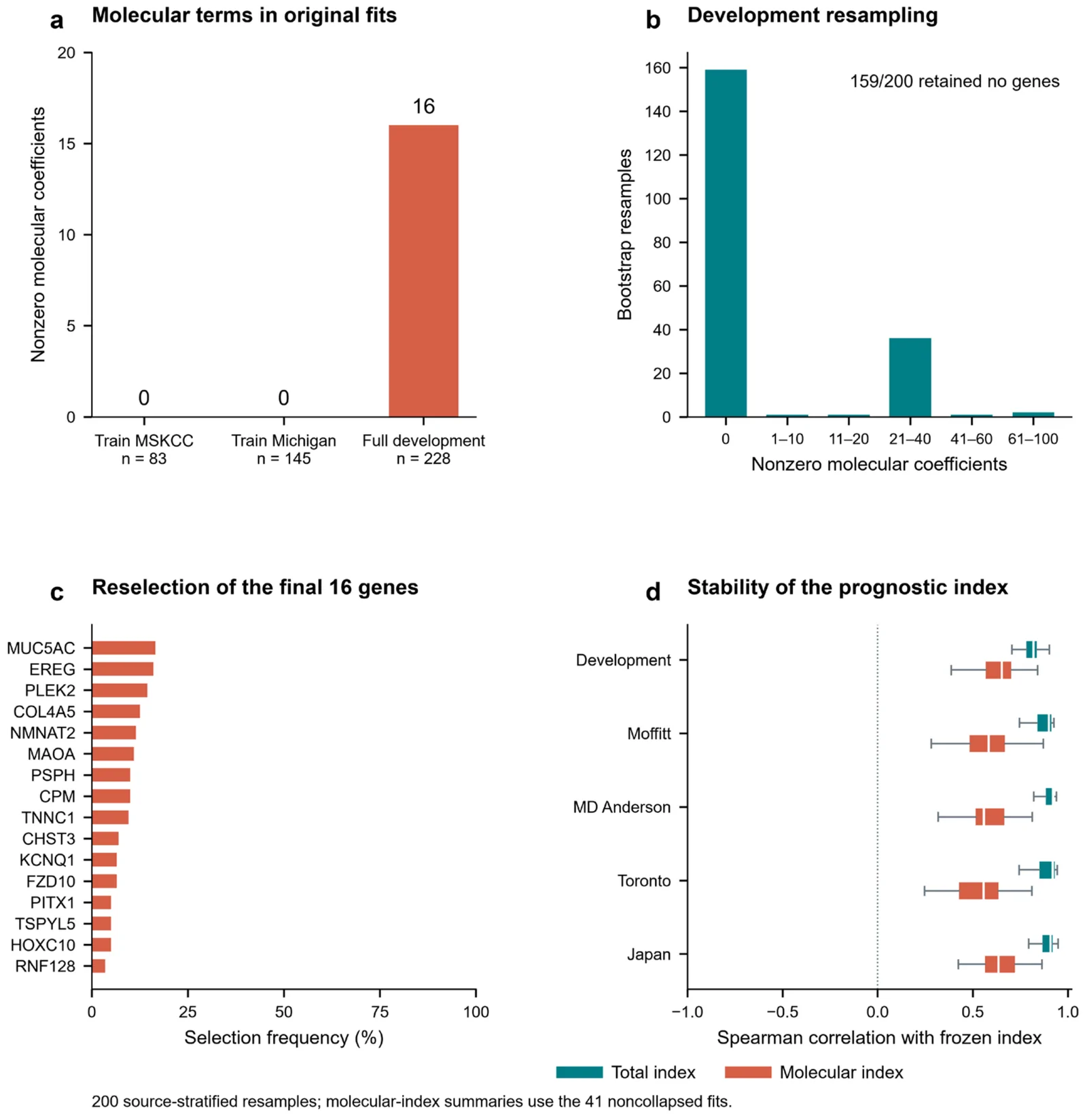
Stability of gene-model learning. (**a**) Nonzero molecular coefficients in two outer source-held-out fits and full-development fit. (**b**) Counts from 200 source-stratified development bootstrap resamples, each repeating filtering, tuning and fitting; 159 retained no genes. (**c**) Reselection frequencies of all 16 genes in final model, using all 200 resamples as denominator. (**d**) Spearman correlations of resampled versus final total and molecular-only indices in each original cohort. Boxes span 25th–75th percentiles; lines mark medians; whiskers extend to observations within 1.5 interquartile ranges. Outliers are omitted from display. Total-index summaries use 200 fits, and molecular-index summaries use 41 noncollapsed fits. These distributions describe development-sample sensitivity rather than external performance uncertainty.

Across 200 development resamples, penalties 0.005, 0.015, 0.05, 0.15 and 0.5 were selected 0, 0, 2, 38 and 160 times, respectively. Of 160 fits selecting 0.5, 159 retained no genes, and one retained one. Fits selecting 0.15 retained a median of 29 genes (range 19–43); the two selecting 0.05 retained 84 and 95. Overall, 159/200 fits collapsed, and final-gene reselection frequencies were 3.5–16.5%. Median external total-index correlations were 0.90–0.92, versus 0.56–0.64 for molecular-only indices among the 41 noncollapsed fits. The more stable total indices were largely supported by the clinical component (Figure 2; Tables S15, S17 and S26).

### 3.3. Frozen-Model Increments Varied Across External Cohorts

**Figure 3:**
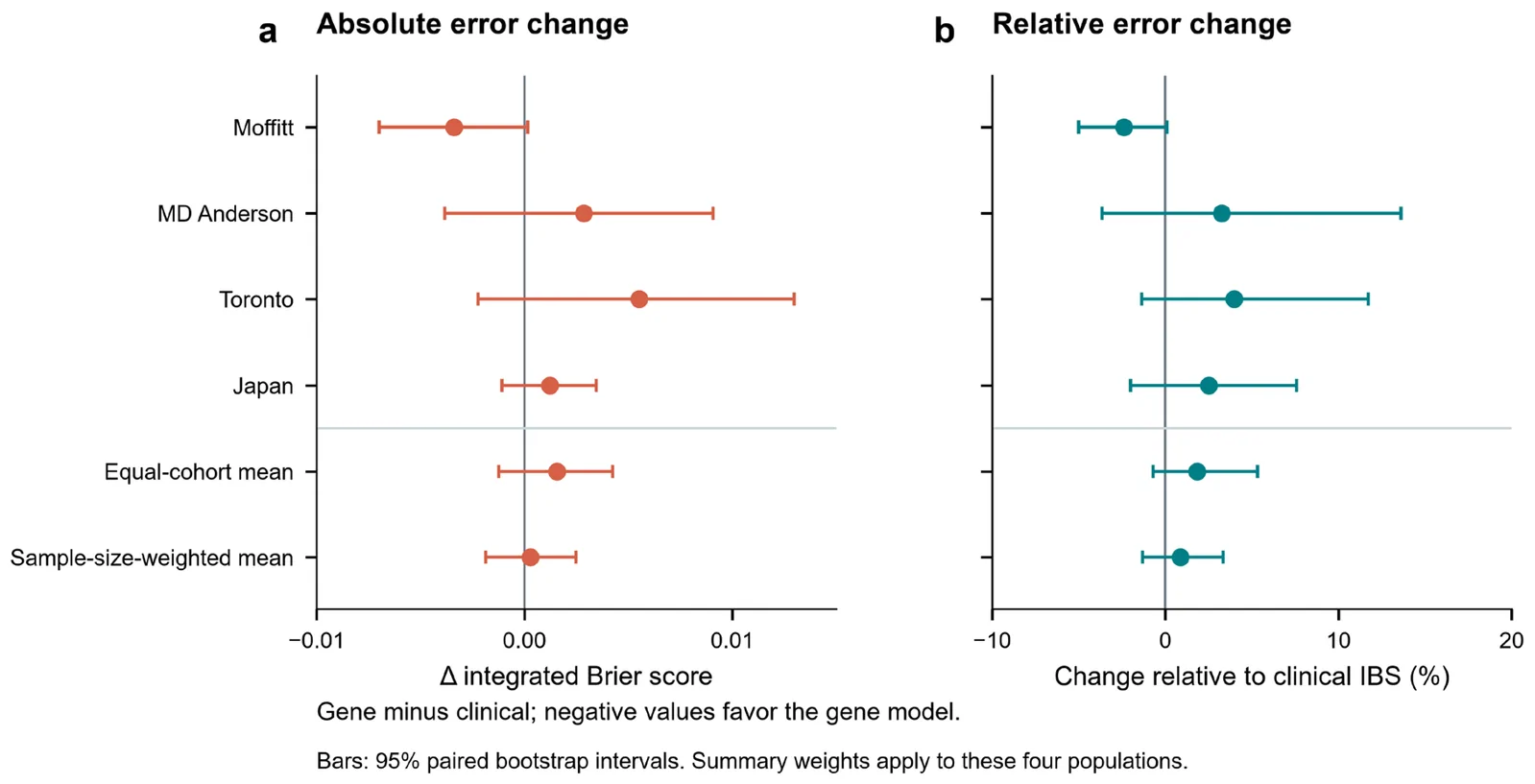
The cohort-specific incremental prediction error of the frozen gene model. (**a**) Absolute gene-minus-clinical IBS differences over 1–3 years. (**b**) Relative changes, 100 × ΔIBS/clinical IBS, calculated within cohorts. Negative values favor the gene model. Bars show percentile 95% intervals from 2000 paired bootstrap resamples per cohort with censoring weights recomputed. Summary rows use equal cohort or sample size weights across the four specified populations; relative changes are calculated before weighting. Intervals condition on the frozen models. IBS, integrated Brier score.

Frozen gene-model ΔIBS was −0.0034 in Moffitt, 0.0029 in MD Anderson, 0.0055 in Toronto and 0.0012 in Japan (Figure 3; Table 3). Relative to clinical IBS, these corresponded to −2.40%, 3.25%, 3.97% and 2.51%. All four 95% intervals included zero. The pathway model’s molecular collapse produced clinical-like error (Table S6). These small, directionally heterogeneous contrasts favor cohort-specific interpretation over a single pooled judgment.

The gene model’s equal cohort mean ΔIBS was 0.0016 (95% CI, −0.0012 to 0.0042), compared with a sample-size-weighted mean of 0.0003 (−0.0019 to 0.0025). Equal cohort and sample-size-weighted relative error changes were 1.83% (−0.70% to 5.32%) and 0.86% (−1.31% to 3.35%), respectively. Thus, weighting affected the point estimate, while both summaries remained compatible with gains or losses. Descriptive heterogeneity was Q = 7.03 on 3 degrees of freedom (*p* = 0.071), with I^2^ = 57.3%; four cohorts provided limited precision for characterizing heterogeneity (Table S18).

Forest and boosting equal cohort ΔIBS values were 0.0024 (95% CI, −0.0015 to 0.0065) and 0.0037 (−0.0005 to 0.0081). Adding pathways to the corresponding clinical-only implementation increased IBS in three cohorts for forest and all four for boosting (Table S12). These findings concern the prespecified grids and implementations, not the attainable performance of either algorithm family.

### 3.4. Locally Updated Baselines Changed the Incremental Comparison

**Figure 4:**
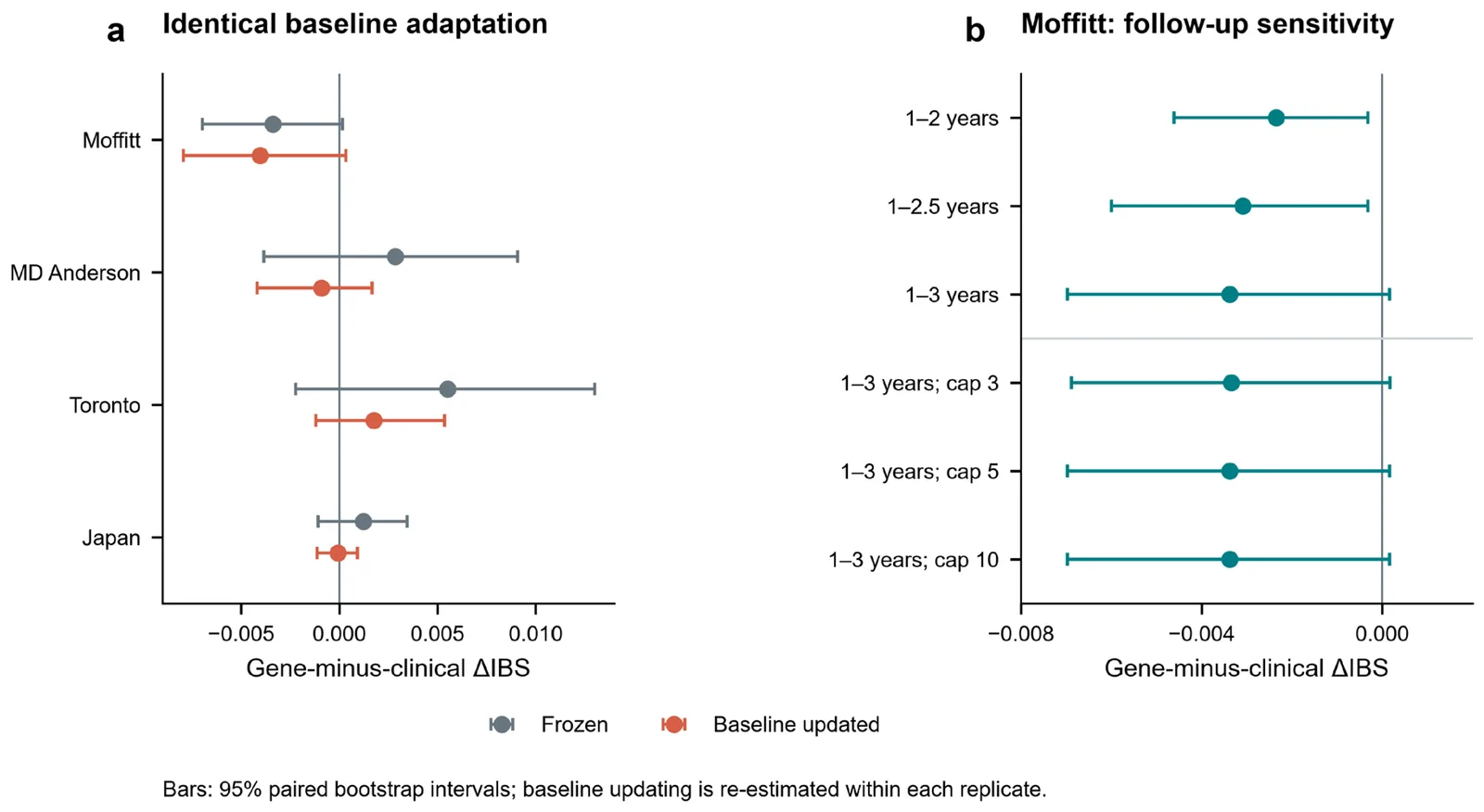
Baseline adaptation and censoring sensitivity. (**a**) Paired gene-minus-clinical IBS contrasts before and after identical five-fold cross-fitted baseline updating. The updated-model intervals re-estimate both baselines within each of 2000 paired bootstrap replicates, keeping all copies of a resampled patient in the same original fold and recomputing censoring weights. (**b**) Moffitt contrasts over shorter horizons and with capped 1–3-year inverse censoring weights. Caps are applied to each weight before scoring. All bars are percentile 95% intervals; negative differences favor the gene model. These analyses are exploratory.

After identical cross-fitted local baseline updating, gene-minus-clinical ΔIBS was −0.0040 in Moffitt, −0.0009 in MD Anderson, 0.0018 in Toronto and −0.0001 in Japan (Figure 4a; Table 4). MD Anderson and Japan shifted from higher frozen gene-model error to slightly lower updated error. Each cohort-specific interval included zero.

The equal cohort mean ΔIBS was −0.0008 (95% CI, −0.0024 to 0.0007), compared with 0.0016 for complete frozen models; the sample-size-weighted updated mean was −0.0015 (−0.0033 to 0.0004). The original frozen and locally updated comparisons describe complementary prediction procedures. Their difference demonstrates sensitivity to local baseline estimation, without uniquely attributing error to baseline-risk transport or molecular information.

### 3.5. Censoring Sensitivity and Exploratory Horizon Effects

Moffitt had median follow-up of 2.49 years, G(3) = 0.266 and 60 patients still under observation at 3 years. At that horizon, 129 patients made nonzero IPCW outcome contributions; the maximum weight was 3.76, and the effective sample size was 101.5 or 32.1% of the original cohort. At 2 years, the effective sample size was 253.8 (80.3%), and the maximum weight was 1.28 (Table S19).

In Moffitt, gene-minus-clinical ΔIBS was −0.0024 (95% CI, −0.0046 to −0.0003) over 1–2 years and −0.0031 (−0.0060 to −0.0003) over 1–2.5 years (Figure 4b; Table S20). The overlapping windows and multiplicity-unadjusted intervals provide an exploratory within-cohort signal, not an independent replication of an early molecular benefit. Capping 1–3-year IPCWs at 3 gave a ΔIBS of −0.0033 (−0.0069 to 0.0002), close to the uncapped result.

With smoking added to the clinical benchmark, gene-model ΔIBS was −0.0033 (95% CI, −0.0069 to 0.0004) in Moffitt, 0.0056 (−0.0024 to 0.0128) in Toronto and 0.0017 (−0.0006 to 0.0040) in Japan (Table S7). Smoking was unavailable in MD Anderson; training-derived imputation addressed missing values where the variable was recorded. Gene-model stage I comparisons retained the original cohort-specific directions, and the exclusion of 22 Japanese records did not produce consistent molecular improvement (Table S8). Censoring tie conventions changed individual-model IBS by less than 0.00003 (Table S13); all 1200 batch recomputations preserved within-patient ranks.

### 3.6. Calibration and Model-Assumption Diagnostics

**Figure 5:**
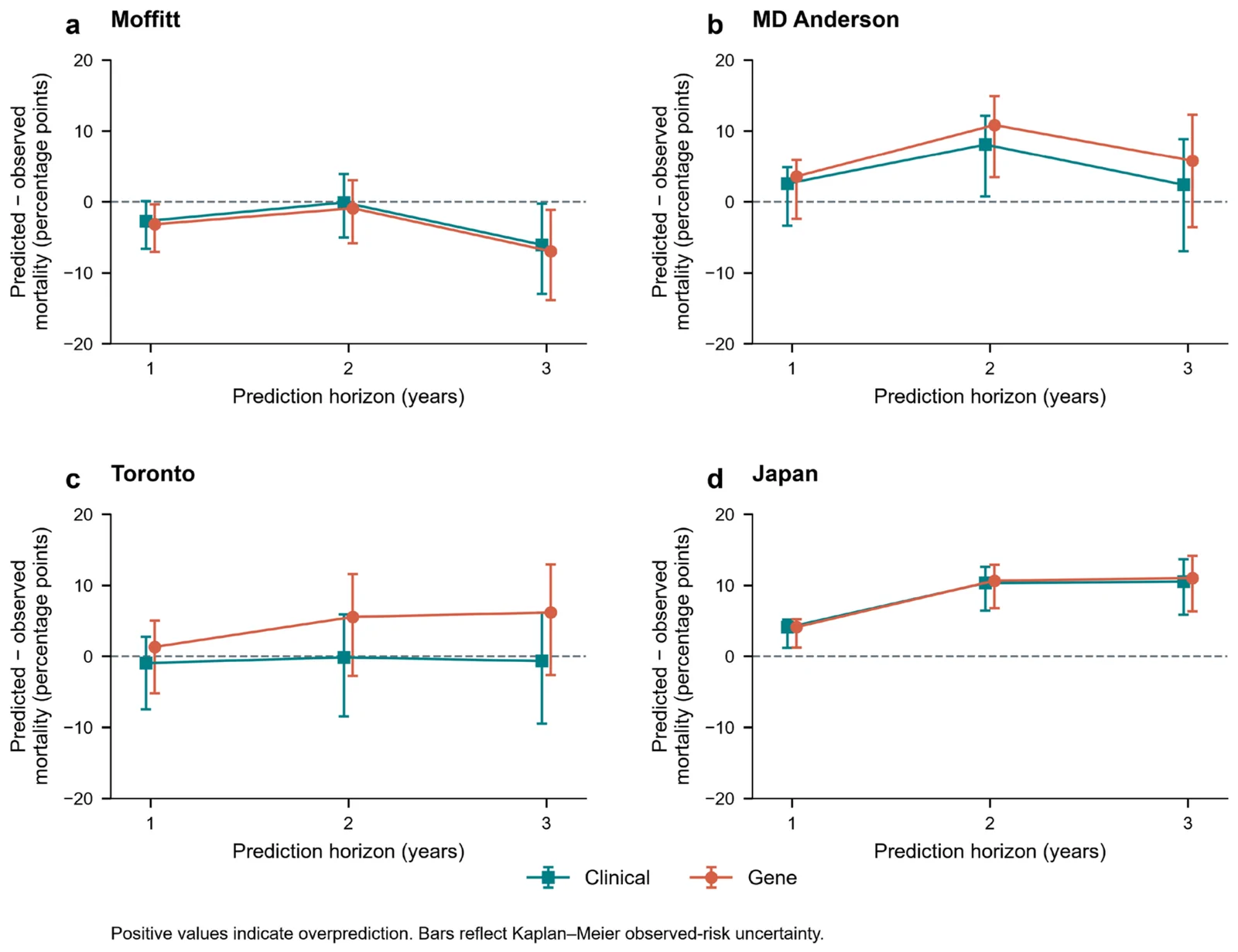
Calibration across prediction horizons. Panels show (**a**) Moffitt, (**b**) MD Anderson, (**c**) Toronto and (**d**) Japan. Points are mean predicted minus Kaplan–Meier observed mortality at 1, 2 and 3 years for the frozen clinical and gene models. Positive values indicate overprediction. Vertical intervals transform the 95% Kaplan–Meier interval for observed mortality while treating mean predictions as fixed; they are not full confidence intervals for calibration error. Symbols are offset horizontally for readability. The dashed line denotes the agreement of mean predicted and observed risks. Table S21 also reports horizon-specific calibration slopes.

Calibration showed substantial population-level mismatch despite modest incremental IBS contrasts. At 3 years, the clinical model predicted a mortality of 19.6% in Japan versus 9.1% observed and 22.6% in Moffitt versus 28.6% observed. The corresponding gene-model predictions were 20.1% and 21.7%. Across cohorts, clinical and gene Uno C values ranged from 0.611 to 0.797 and from 0.604 to 0.761, respectively; 3-year AUC and calibration slopes are reported together in Table 5. Calibration differences were also present at earlier horizons (Figure 5; Table S21). Risk-tertile displays and their support appear in Figure S2 and Table S14.

Frozen-index proportional-hazards tests yielded nominal *p* values of 0.013 and 0.010 for the clinical and gene models in Japan and 0.048 for the gene model in MD Anderson. No proportional-hazards diagnostic had a false-discovery-rate-adjusted q value below 0.05 (Table S22). The nominal signals motivate attention to time-varying effects, while the modest event counts limit diagnostic sensitivity. Age spline knots were 51, 65 and 77 years. The development clinical model’s nonlinear-age likelihood-ratio test had *p* = 0.059; all age tests had q ≥ 0.42 (Table S23).

### 3.7. Molecular Retention and Performance Depended on Specification

Changing the gene model’s mixing fraction to 0.1 or 0.9 retained 61 or 1 gene, respectively; filtering to 100 or 1000 candidates retained 7 or 0. A denser penalty grid selected 0.25 and retained one gene. Pathway models with mixing fractions 0.1 and 0.9 retained four and five pathways, while the original and denser-grid models retained none. Five pathway principal components explained 69.5% of training variance. No assessed specification had lower point-estimate IBS than the clinical model in all four cohorts. Exploratory intervals favored the 100-gene-filter model in Moffitt, the 0.1-mixing pathway model in MD Anderson and the five-component model in Japan; each had mixed results elsewhere. Thus, complete molecular loss depended partly on specification, while external increments remained population-dependent (Figure S3; Tables S28 and S29).

## 4. Discussion

Selection instability was the central constraint on the evaluated transcriptomic procedures. Most development resamples selected no genes, and alternative specifications materially changed molecular retention. The original 16-gene model nevertheless remains informative as the frozen output of a defined procedure: its external evaluation measures what that particular fitted rule transfers, while resampling reveals how uncertain its derivation was. Together, these analyses support a limited conclusion about these procedures and this development sample, rather than a claim that transcriptomic measurements lack prognostic information.

The benchmark defines the incremental question. Age, sex and broad historical stage were consistently available, whereas smoking, detailed pathological features, performance status, comorbidity and postoperative treatment were incomplete. Smoking-adjusted results remained directionally heterogeneous. A richer clinical model could absorb information currently represented by expression or change the remaining molecular increment through different risk relationships. These data do not determine its direction or magnitude. Clinical utility would additionally require a decision context and the consequences of acting on predicted risk.

Limited development information plausibly contributed to unstable selection. Cox estimation used 96 deaths, but the primary 3-year prediction horizon contained only 46. Source-based tuning summarized just two transfer contrasts, with unequal source sizes and mortality. The full-development preference for penalty 0.15 over 0.5 rested on a small score difference; bootstrap repetitions usually favored stronger penalization. Repeated patient sampling does not create additional independent source-level experience. This combination limits the reliable identification of a high-dimensional molecular component, consistent with evidence that shrinkage parameters and predictions remain uncertain in small samples [9,10,11,12]. The 16 retained genes should not be interpreted as having overcome the original candidate-selection burden.

An absolute IBS difference of 0.001–0.005 is a small change in average squared prediction error on a 0–1 scale, not a 0.1–0.5 percentage-point change in mortality or a treatment benefit. Relative error changes were also modest and varied by cohort. No clinically meaningful IBS threshold or treatment decision rule was specified, so statistical uncertainty and practical relevance require separate judgments. Cohort-specific estimates remain the inferential focus; equal cohort and sample-size-weighted summaries describe these selected populations and do not estimate performance across contemporary institutions.

The clearest evidence of limited absolute-risk transport was the calibration mismatch in Japan and Moffitt. Locally updating both baseline hazards changed the incremental comparison while preserving the original linear predictors. This is useful evidence about adaptation when local outcome data are available [6,7], but it is neither complete-model transport nor a unique decomposition of error. Baseline estimation interacts with covariate effects, case mix, censoring and possible model misspecification. Cross-fitting separates local estimation from evaluation, and bootstrap refitting reflects that estimation uncertainty. It cannot establish which mechanism caused the original miscalibration.

Transport was evaluated across known compatible assays. Evaluation-platform annotations helped define the 10,029-gene intersection and pathway coverage, so outcome independence did not make the feature definition independent of the evaluation platforms. Deployment to an unseen assay would require a development-defined panel, a missing-feature policy and separate measurement validation. The intersection excluded E2F targets and MYC targets V2, while historical array normalization remained part of the input data. These conditions restrict both the biological representation and the transport setting examined.

This study complements multisite expression-based prediction in the Director’s Challenge [1] and prospective clonal-biomarker evaluation in ORACLE [2] by examining uncertainty in a specific public-data learning procedure. The ISRM comparator similarly evaluates a refitted gene set, rather than its published score [3]. Biological coherence supplies a different kind of evidence: work on the abscopal effect and systemic immune reprogramming distinguishes molecular and immune changes from demonstrated clinical benefit [32]. The descriptive RNA–protein analysis in the Supplementary Materials addresses the covariance of pathway measurements [33]; prognostic utility is assessed by the survival comparisons.

We selected historical surgical datasets with heterogeneous stage editions, treatment recording, resection completeness, follow-up and baseline mortality. Moffitt’s effective sample size fell to approximately 102 at 3 years; shorter horizons improve censoring support, but the overlapping exploratory findings do not establish reproducible early benefit. Marginal IPCW relies on independent censoring, an assumption not tested by weight truncation. Patient identities and surgical dates could not be conclusively linked across studies. Modern adjuvant therapies further limit the direct interpretation of these historical absolute risks [34]. A subsequent evaluation should use a justified development sample across more sources, a fixed assay, richer clinical predictors and a separate assessment of frozen and locally adapted predictions.

## 5. Conclusions

In four selected historical cohorts of resected lung adenocarcinoma, the evaluated transcriptomic models showed unstable molecular selection and inconsistent incremental prediction beyond age, sex and broad pathological stage. Alternative specifications changed molecular retention, and local baseline updating changed prediction-error contrasts. These findings characterize the limitations of the assessed learning procedures and development sample; broader transcriptomic prognostic value remains unresolved, and contemporary clinical utility requires separate evaluation.

## Figures and Tables

**Table 1:** The characteristics of the development and external cohorts.

Characteristic	Development	Moffitt	MD Anderson	Toronto	Japan
Eligible patients	228	316	109	127	226
Observed deaths	96	76	31	51	35
Deaths by 3 years	46	69	18	29	20
Age, median (IQR), years	65.0 (58.8–73.0)	70.0 (64.0–77.0)	64.6 (58.1–72.3)	69.9 (62.8–75.7)	61.0 (55.0–65.0)
Male, *n* (%)	105 (46.1%)	145 (45.9%)	52 (47.7%)	65 (51.2%)	105 (46.5%)
Stage I, *n* (%)	179 (78.5%)	249 (78.8%)	87 (79.8%)	92 (72.4%)	168 (74.3%)
Stage II, *n* (%)	49 (21.5%)	67 (21.2%)	22 (20.2%)	35 (27.6%)	58 (25.7%)
At risk at 3 years	166	60	70	84	185
Censoring survival at 3 years	0.906	0.266	0.790	0.876	0.901
Median follow-up, years	5.83	2.49	5.20	5.49	5.06
Smoking status missing, *n* (%)	70 (30.7%)	50 (15.8%)	Not recorded	12 (9.4%)	0 (0.0%)
Adjuvant chemotherapy, *n* (%)	32 (14.0%)	Not recorded	28 (25.7%)	Not recorded	Not recorded
Adjuvant status missing, *n* (%)	5 (2.2%)	Not recorded	0 (0.0%)	Not recorded	Not recorded

Values describe eligible patients. IQR, interquartile range. Development used only Michigan and Memorial Sloan Kettering sources of GSE68465. Follow-up was estimated by reverse Kaplan–Meier. Censoring survival is the probability of remaining uncensored. Broad historical stage groups were retained; contemporary TNM stages were not reconstructed.

**Table 2:** The molecular coefficients and reselection frequencies of the frozen 16-gene model.

Gene	Standardized Coefficient	Selected/200	Reselection, %
MUC5AC	0.108184	33	16.5
EREG	0.075208	32	16.0
RNF128	0.000518	7	3.5
HOXC10	−0.007009	10	5.0
PSPH	0.046230	20	10.0
NMNAT2	−0.048888	23	11.5
TNNC1	−0.041478	19	9.5
COL4A5	−0.077021	25	12.5
KCNQ1	−0.019060	13	6.5
CHST3	0.053911	14	7.0
TSPYL5	−0.007643	10	5.0
MAOA	−0.026162	22	11.0
FZD10	−0.008123	13	6.5
PITX1	0.018988	10	5.0
PLEK2	0.108383	29	14.5
CPM	−0.009290	20	10.0

Coefficients apply to training-standardized within-patient gene ranks. Reselection uses all 200 development resamples, including 159 collapsed fits. Tables S11, S24 and S25 provide scaling, clinical coefficients, centering offsets and baseline survival.

**Table 3:** Frozen clinical and gene-model prediction error over 1–3 years.

Population/Summary	Clinical IBS	Gene IBS	Gene–Clinical ΔIBS (95% CI)	Relative Change, % (95% CI)
Moffitt	0.1409	0.1376	−0.0034 (−0.0070, 0.0002)	−2.40 (−5.01, 0.11)
MD Anderson	0.0879	0.0907	0.0029 (−0.0038, 0.0091)	3.25 (−3.66, 13.59)
Toronto	0.1392	0.1447	0.0055 (−0.0022, 0.0130)	3.97 (−1.36, 11.72)
Japan	0.0491	0.0503	0.0012 (−0.0011, 0.0035)	2.51 (−2.01, 7.58)
Equal cohort mean	0.1043	0.1058	0.0016 (−0.0012, 0.0042)	1.83 (−0.70, 5.32)
Sample size mean	0.1065	0.1068	0.0003 (−0.0019, 0.0025)	0.86 (−1.31, 3.35)

Negative contrasts favor the gene model. The benchmark comprises age, sex and broad pathological stage. Intervals use 2000 paired patient bootstrap resamples per cohort, conditional on the frozen models. Equal cohort weights are 25% each; sample size weights are n/778. Relative contrasts are calculated within cohorts before weighting. The primary pathway model retained no molecular terms; all model-family estimates appear in Table S6.

**Table 4:** Incremental gene-model error after identical cross-fitted baseline updating.

Population/Summary	Updated Clinical IBS	Updated Gene IBS	Updated ΔIBS (95% CI)	Frozen ΔIBS
Moffitt	0.1408	0.1368	−0.0040 (−0.0079, 0.0003)	−0.0034
MD Anderson	0.0871	0.0862	−0.0009 (−0.0042, 0.0017)	0.0029
Toronto	0.1431	0.1449	0.0018 (−0.0012, 0.0054)	0.0055
Japan	0.0450	0.0449	−0.0001 (−0.0011, 0.0009)	0.0012
Equal cohort mean	0.1040	0.1032	−0.0008 (−0.0024, 0.0007)	0.0016
Sample size mean	0.1058	0.1043	−0.0015 (−0.0033, 0.0004)	0.0003

Both models use the same local five-fold partition. Each of 2000 bootstrap replicates resamples patients within their original folds, re-estimates both baseline hazards and recomputes censoring weights. Intervals condition on the frozen coefficients and original fold allocation. Negative differences favor the gene model. Summary absolute IBS values use the stated cohort weights.

**Table 5:** Discrimination and 3-year calibration of frozen clinical and gene models.

Cohort	Model	Uno C	3-Year AUC	Predicted Death, %	Observed Death, %	Calibration Slope (95% CI)
Moffitt	Clinical	0.616	0.607	22.6	28.6	0.85 (0.36, 1.35)
Moffitt	Gene	0.630	0.639	21.7	28.6	0.96 (0.53, 1.40)
MD Anderson	Clinical	0.651	0.636	21.0	18.6	0.93 (0.06, 1.80)
MD Anderson	Gene	0.665	0.655	24.4	18.6	0.97 (0.18, 1.77)
Toronto	Clinical	0.611	0.633	23.8	24.5	0.88 (0.11, 1.66)
Toronto	Gene	0.604	0.632	30.7	24.5	0.71 (0.03, 1.39)
Japan	Clinical	0.797	0.807	19.6	9.1	2.46 (1.45, 3.48)
Japan	Gene	0.761	0.772	20.1	9.1	1.76 (0.97, 2.56)

Uno C is truncated at 3 years; AUC is cumulative/dynamic at 3 years. Observed mortality uses Kaplan–Meier estimation. Calibration slopes use the frozen log cumulative predicted hazard with follow-up truncated at 3 years; intervals are model-based. These discrimination point estimates complement the paired prediction-error contrasts in Table 3.

## Data Availability

The expression and clinical data are publicly available from GEO (https://www.ncbi.nlm.nih.gov/geo/) under GSE68465, GSE72094, GSE42127, GSE50081 and GSE31210; platform annotations are GPL96, GPL15048, GPL6884 and GPL570. Hallmark gene sets were obtained from MSigDB release 2025.1.Hs (https://www.gsea-msigdb.org/gsea/msigdb/). The harmonized CPTAC matrices are available at https://doi.org/10.5281/zenodo.8394329, and the original proteomic study is accessible through the NCI Proteomic Data Commons (PDC000153). Analysis summaries are provided in the manuscript and Supplementary Materials. Supplementary Methods S2 defines the gene universe and pathway measurements; Tables S11, S24 and S25 report Cox coefficients, centering offsets and baseline survival functions through 3 years. This study generated no new raw sequencing or proteomic data.

## Supplementary Materials

The following supporting information can be downloaded at https://www.mdpi.com/article/10.3390/cancers18203361/s1, Supplementary Methods S1–S8, Table S1: Endpoint provenance and harmonization; Table S2: Sequential eligibility accounting; Table S3: Hallmark coverage on the known four-platform gene universe; Table S4: Prespecified model grids and selected final settings; Table S5: Nested source-held-out performance and molecular selection; Table S6: Complete frozen external performance; Table S7: Smoking-extended clinical comparison; Table S8: Stage I and original prognosis-eligible comparisons; Table S9: Paired incremental error after cross-fitted baseline updating; Table S10: RNA–protein pathway covariance; Table S11: The clinical and nonzero molecular coefficients of the frozen gene model; Table S12: Clinical-only and pathway models within the same algorithm; Table S13: Censoring tie convention; Table S14: Three-year calibration-group support and transported cutpoints; Table S15: Development bootstrap selection summary; Table S16: Gene reselection frequencies; Table S17: Correlation with the frozen gene-model prognostic index; Table S18: Complementary cohort aggregation; Table S19: Censoring-weight support across horizons; Table S20: Follow-up horizon and capped-weight sensitivity; Table S21: Calibration at 1, 2 and 3 years; Table S22: Proportional-hazards diagnostics through 3 years; Table S23: Nonlinear-age diagnostics through 3 years; Table S24: The clinical coefficients and centering offsets of the frozen Cox models; Table S25: Frozen baseline survival functions through 3 years; Table S26: Joint distribution of selected penalty and retained gene counts across 200 development resamples; Table S27: Original gene-model tuning scores for final source-based selection; Table S28: Development-only selection for alternative modeling specifications; Table S29: Cohort-specific external performance of all assessed specifications; Table S30: A prespecified equal-weight summary across the two US evaluation cohorts; Figure S1: RNA–protein pathway concordance in public CPTAC tumors; Figure S2: Calibration of frozen models at 3 years; Figure S3: Dependence of external prediction error on modeling specification. Supplementary File S1: Internally timestamped analysis specification. The descriptive proteomic methods and results are reported in Supplementary Methods S7, Table S10 and Figure S1 [33].
