# Supplementary material for: Selection Instability and External Performance of Transcriptomic Models Beyond Age, Sex and Stage in Resected Lung Adenocarcinoma
Source: Cancers (Basel). 2026 Oct 17;18(20):3361. doi: 10.3390/cancers18203361 (PMC13702175; doi:10.3390/cancers18203361)
Supplement: Supplementary file 1 [file cancers-18-03361-s001.zip › cancers-4599405 - Supplementary File S1.pdf]

Supplementary File S1. Internally Timestamped Analysis Specification

Provenance

The following record reproduces the archived analysis specification. The recorded UTC timestamp is 2026-09-15T08:24:20.680549+00:00. This is a local record rather than a prospectively registered protocol or an independently certified timestamp. This document presents the archived content; it does not assign the original timestamp to analyses added later.

SHA-256 of the archived specification:
ad9c8fc9d3aa6f235b07bf7d41d9996351a7480a7e3acb857479589cbb1ba978

The original model-freeze record is dated 2026-09-15T08:28:19.904337+00:00 and records model SHA-256 37c85e7fe6180a8ce47b9b595cc703624cfaf2e438286867f7f86ee8c007ac65. The analysis outputs retain the original frozen models.

Archived specification

Created utc: 2026-09-15T08:24:20.680549+00:00

Status: Analysis specification frozen before fitting or external performance inspection; metadata and endpoint counts may be inspected for feasibility. Not prospectively registered.

Primary population: Adult stage I-II surgically resected lung adenocarcinoma with valid overall survival

Development: GSE68465 MI and MSKCC only; HLM and pooled DFCI/CAN excluded to reduce overlap with validation centers

External: ["GSE72094", "GSE42127", "GSE50081", "GSE31210"]

Tcga: Diagnosis-origin survival prevents pooling with surgical time origin. RNA-seq extension is exploratory discrimination only if feasible; no postoperative absolute-risk claim.

Features: Intersection of valid single-symbol probes on four platforms; median probes per gene; within-sample ranks; Hallmark coverage >=80% and >=10 genes; no external outcome-based filtering.

Clinical: ["age", "male", "stage_II"]

Primary model: Clinical plus Hallmark pathway Cox elastic net (l1_ratio 0.5), clinical coefficients unpenalized

Secondary models: ["clinical plus top 500 variance-ranked genes Cox elastic net", "clinical plus pathway random survival forest", "clinical plus pathway gradient-boosted Cox", "clinical plus five ISRM genes elastic-net refit"]

Tuning: Leave-one-development-site-out for final selection, equal-site mean IBS; nested leave-site-out with inner three-fold event-stratified CV for internal validation; every imputer, scaler, variance filter fitted in training fold.

Cox alphas: [0.005, 0.015, 0.05, 0.15, 0.5]

Clinical penalty: 0.01

Trees: 200

Forest leaf: [10, 20]

Boosting grid: [{"depth": 1, "learning_rate": 0.03}, {"depth": 1, "learning_rate": 0.1}, {"depth": 2, "learning_rate": 0.03}]

Primary estimand: Paired difference in external IPCW integrated Brier score over 1-3 years, pathway model minus clinical; equal weight across four cohorts, and US-only average

Ipcw: Censoring estimated within each evaluated cohort, re-estimated within each patient bootstrap; event weights use G(T-)

Uncertainty: 2000 paired within-cohort bootstrap replicates, percentile 95% confidence intervals, conditional on the frozen model; equal-cohort mean uses independent within-cohort resampling

Secondary metrics: ["Uno C truncated at 3 years", "cumulative/dynamic AUC at 3 years", "calibration slope over follow-up truncated at 3 years", "KM observed and mean predicted 3-year mortality"]

External rules: No external fitting, scaling, batch correction or optimized cutpoints in primary analysis. Do not select best model by external performance.

Sensitivity: ["clinical plus smoking with foldwise imputation where available", "GSE31210 excludes original incomplete resection/adjuvant-treatment flag", "stage I only", "3-year survivors risk-set adequacy", "cohort median centering stress test compared with fixed within-patient scores"]

Recalibration: Optional exploratory five-fold cross-fitted baseline update with fixed prognostic index and only intercept/baseline updated; never use same patient to calibrate and evaluate.

Duplicate rule: Exact expression profiles or identical within-study patient IDs reviewed before fitting; cross-study expression correlation is an overlap screen, not proof of absence of duplicate patients.

Clinical use: Prognostic evaluation; no treatment-benefit recommendation, prospective clinical deployment or decision-curve treatment utility claim.

Relationship to the reported analyses

The primary pathway comparison, original model grids, cohort roles and external evaluation rules follow the archived record. The main manuscript centers cohort-specific estimates and retains the originally defined equal-cohort summary. The prespecified US-only summary is provided below. TCGA was excluded from postoperative prediction because its diagnosis-based time origin did not match the surgical target. The optional TCGA discrimination extension was not undertaken.

The detailed development bootstrap, paired bootstrap refitting of locally updated baselines, alternative cohort weighting, expanded censoring diagnostics and functional-form diagnostics were completed after the original model freeze. Alternative mixing fractions, denser penalty grids, gene-filter sizes and pathway principal components are post hoc specification sensitivities. These analyses retain their exploratory status and do not replace the original primary estimand or original fitted models.
