# Supplementary material for: Selection Instability and External Performance of Transcriptomic Models Beyond Age, Sex and Stage in Resected Lung Adenocarcinoma
Source: Cancers (Basel). 2026 Oct 17;18(20):3361. doi: 10.3390/cancers18203361 (PMC13702175; doi:10.3390/cancers18203361)
Supplement: Supplementary file 1 [file cancers-18-03361-s001.zip › cancers-4599405 - Supplementary Methods, Figures and Tables.pdf]

Supplementary Materials

Selection Instability and External Performance of Transcriptomic Models Beyond Age, Sex and Stage in Resected Lung Adenocarcinoma

Zhihao Lei, Chenxi Zou and Wei Zhao

Supplementary Methods S1. Cohorts and endpoint harmonization

GEO series GSE68465, GSE72094, GSE42127, GSE50081 and GSE31210 provided processed expression and clinical metadata. Compressed-file integrity and checksums were verified. Development was restricted to the Michigan and Memorial Sloan Kettering sources of GSE68465. Excluding its Moffitt and pooled Dana-Farber/Canadian sources reduced potential institutional overlap with external cohorts. These exclusions were precautionary; they did not imply that duplicate patients had been identified.

Overall survival denoted time from surgical resection to death from any cause or final follow-up, as supported by published cohort descriptions (Table S1). Individual operation dates were unavailable. Months were divided by 12 and days by 365.25. GSE31210 metadata contained a duplicated months field and variable field positions across samples. Each sample was parsed by field name, using explicit days to death/censoring; this field agreed with the second months value multiplied by 30. Normal specimens were excluded by tissue labels. TCGA diagnosis-origin endpoints did not enter the reported prediction analyses.

Histology, broad pathological stage, age, sex and valid follow-up determined eligibility (Table S2). Broad stage retained the historical source definition rather than a retrospectively assigned common TNM edition. Treatment was descriptive: development had 223 known adjuvant-treatment records, including 32 treated patients, and five missing records; MD Anderson had 109 known records, including 28 treated patients. Treatment was not consistently available elsewhere. A separate GSE31210 analysis excluded 22 records flagged for incomplete resection or adjuvant therapy. Smoking extension used ever-smoking status with training-derived imputation where necessary; no smoking information was available in the harmonized MD Anderson metadata.

Supplementary Methods S2. Feature definition and learning procedure

The gene universe comprised 10,029 symbols shared by GPL96, GPL15048, GPL6884 and GPL570. Probe mappings required one unambiguous symbol; multiple probes were summarized by their within-sample median. Evaluation-platform annotations contributed to assay compatibility, without using evaluation outcomes or expression distributions. This outcome-independent, evaluation-platform-informed feature definition assumes knowledge of the measured platforms. A deployment to an unknown future assay would require a separately specified feature and missing-measurement policy.

Ascending within-patient ranks used average ranks for ties. A Hallmark set with k measured genes among N background genes received score $[\text{mean(rank)} - (N + 1)/2]/(N - k)$. MSigDB release 2025.1.Hs supplied pathway membership. The eligibility threshold was at least 10 genes and 80% original membership coverage (Table S3). Fixed ranks were unchanged in 1200 batch-composition recomputations: one fixed patient from each external cohort, 100 resampled batches at sizes 10, 25 and 50. Original array normalization was a separate, study-specific measurement step.

Table S4 lists candidate and selected settings. Each training fold fitted its own median imputer, standardizer and, for the gene model, 500-gene variance filter. Source-separated tuning assessed transfer between Michigan and Memorial Sloan Kettering. Nested internal assessment held out one source and used three-fold event-stratified tuning in the other. Numerically failed or explicitly nonconvergent candidate fits were assigned infeasible scores. Final fitting used the complete development sample; all prediction parameters and baseline survival functions were frozen before evaluation. The main random seed was 20260915.

The pathway and immunosenescence refit models set all molecular coefficients to zero. Their small differences from the clinical reference arise from the ridge penalty on the reference's clinical coefficients and numerical implementation. They should not be interpreted as precise evidence of molecular equivalence.

Both outer-fold gene models also retained zero molecular coefficients (Table S5). The final gene model's nonzero molecular coefficients and clinical coefficients are in Table S11; coefficients apply to training-standardized features and are prognostic parameters rather than causal effects.

Absolute Cox survival is S0(t) raised to $\exp[\Sigma \beta_j (x_j - \mu_j)/\sigma_j - \text{offset}]$. The baseline S0(t) is a right-continuous step function, equal to one before its first listed time. Table S11 reports all nonzero coefficients and their training means and standard deviations for the gene model. Table S24 reports the clinical coefficients and centering offsets for all four Cox models; Table S25 gives their baseline survival functions through 3 years. All remaining molecular coefficients are zero. The gene universe and pathway scores follow the platform-intersection and rank definitions above. Calculations used unrounded parameters.

Supplementary Methods S3. Prediction error, aggregation and censoring

At evaluation time t, a death at $T \leq t$ contributes $S(t)^2/G(T-)$, a patient observed beyond t contributes $[1 - S(t)]^2/G(t)$, and an earlier censored observation contributes zero. Contributions are averaged over all evaluated patients. S is predicted survival and G is the evaluated cohort's Kaplan–Meier censoring-survival estimate. The primary convention retains deaths tied with censoring in the denominator. An event-first reverse-risk convention was assessed separately (Table S13). IBS is the trapezoidal integral over 25 equally spaced times from 1 to 3 years, divided by the two-year width.

For each model comparison, bootstrap resampling was paired across predictions from the same patients and recomputed G. Frozen-model intervals used 2000 replicates per cohort. The four-cohort equal-weight estimand was one quarter of the sum of population-specific contrasts. Sample-size weighting used 316/778, 109/778, 127/778 and 226/778 for Moffitt, MD Anderson, Toronto and Japan. Relative contrasts divided each cohort's ΔIBS by its own clinical IBS before averaging; the denominator was recomputed in each bootstrap draw. These are relative error changes, not Brier skill scores against a null model. Independent draws across cohorts generated summary intervals. Q used inverse bootstrap-variance weights, and $I^2 = \max[0, (Q - 3)/Q] \times 100\%$. With four selected cohorts, heterogeneity estimates are descriptive (Table S18).

IPCW support was summarized at 1, 2, 2.5 and 3 years (Table S19). Weight quantiles refer to nonzero contributions. Effective sample size equals $(\Sigma w)^2/\Sigma w^2$, including zero contributions in the evaluated sample. It is distinct from the risk set: deaths before the horizon contribute known outcomes, while patients censored before it do not. Shorter-horizon IBS used the corresponding subset of the original grid, with 13 points over 1–2 years and 19 over 1–2.5 years. Capping at 3, 5 or 10 replaced each weight by its minimum with the cap; this deliberately modifies the marginal-IPCW estimator. Each configuration used 2000 paired resamples. The same patient-resampling stream was used as in primary evaluation, ensuring that the uncapped 1–3-year intervals agree with the primary analysis. Horizon and truncation intervals are unadjusted for multiplicity. These exploratory comparisons do not establish independent censoring.

Supplementary Methods S4. Development stability

Two hundred patient bootstrap samples preserved the original development-source sizes: 145 Michigan and 83 Memorial Sloan Kettering observations per draw. Sampling with replacement occurred independently within sources. Each draw repeated variance filtering, source-based penalty tuning, preprocessing and final gene-model fitting. All copies of a person remained in the same source during tuning. Tables S15–S17 summarize nonzero counts, gene selection and index stability.

Selection frequency is the proportion of completed development resamples retaining a nonzero coefficient for a gene. It reflects the combined variance-screening and penalized-selection procedure. Jaccard overlap with the final 16-gene set is the number of shared selected genes divided by the size of their union. Spearman correlations compare resampled and final indices in the original patient sets, separately for the total predictor and its molecular-only component. Molecular correlation is undefined for a collapsed model. These distributions characterize the sensitivity of the fitted gene procedure to development-sample composition; they do not constitute independent evaluation of a newly selected model or confidence intervals for performance in future hospitals.

Supplementary Methods S5. Baseline updating and calibration

Within each evaluation cohort, an event-stratified five-fold split was shared by the clinical and gene models. For each held-out fold, baseline hazard increments were fitted in the remaining folds as the number of deaths at a time divided by the sum of exp(frozen prognostic index) in that risk set. The held-out patient's updated survival was $\exp[-H0(t) \times \exp(\text{index})]$. Relative-risk coefficients stayed fixed. Identical splits and training observations made the gene-minus-clinical comparison paired (Table S9).

Each of 2000 bootstrap replicates resampled patients within their original folds, refitted both baseline hazards and recomputed censoring weights. Repeated copies of a patient remained together in their original fold, preventing copies from serving simultaneously as baseline-fitting and evaluation observations. The resulting interval includes local baseline-estimation and evaluation-sample variability conditional on the original models and the chosen fold allocation. Development-selection variability is described separately by Tables S15–S17.

Temporal calibration compared mean predicted mortality with Kaplan–Meier estimates at 1, 2 and 3 years. Mean prediction was treated as fixed in the graphical observed-risk intervals. A horizon-specific calibration slope was estimated from a Cox model containing $\log[-\log S(t)]$ as its single predictor with administrative censoring at t. For a Cox prediction rule this index differs from its linear predictor only by a constant, although the diagnostic slope changes as the event horizon changes. Three-year grouped calibration used development-derived tertiles; Table S14 gives each transported group's sample and follow-up support. Figure S2 displays Kaplan–Meier uncertainty rather than uncertainty from a fitted calibration smoother.

Supplementary Methods S6. Proportional hazards and age form

Global efficient score tests evaluated interactions of the diagnostic covariates with centered log event time. Follow-up was administratively censored at 3 years, and tied deaths used Breslow risk sets. For each cohort and model, one test used the frozen total index; another jointly used age, sex and stage, adding the frozen molecular index for the gene model. Local null-model coefficients were nuisance parameters fitted only for diagnostics.

For covariate vector x and centered log time g(t), the time-interaction score summed g(t) times the observed-minus-risk-weighted covariate totals over event times. Its efficient covariance was $I_{\gamma\gamma} - I_{\gamma\beta} I_{\beta\beta}^{-1} I_{\beta\gamma}$, adjusting for estimated null coefficients. The quadratic score statistic was compared with a chi-square distribution using the covariance rank. Numerical differentiation of the partial-likelihood score independently reproduced all 20 test statistics. These tests assess a specified log-time departure and cannot exclude other forms of nonproportionality. Table S22 reports nominal p values and Benjamini–Hochberg q values across the 20 tests.

Age diagnostics fitted local clinical Cox models, with the frozen molecular index added for the gene comparison. A three-knot restricted cubic spline used the development age percentiles 51, 65 and 77 years. One nonlinear basis term was added to linear age; an Efron-tie partial-likelihood ratio test compared the nested fits on one degree of freedom. No predictor was rebuilt from these diagnostics. Table S23 reports the 10 tests and their separate multiplicity adjustment.

Supplementary Methods S7. Descriptive RNA–protein analysis

The harmonized CPTAC files at https://doi.org/10.5281/zenodo.8394329 supplied BCM lung adenocarcinoma tumor protein abundance, RNA abundance and GENCODE v34 gene mapping. Files were checked against their published MD5 values. Matching by case identifier yielded 106 tumors with usable paired measurements, a subset of the original study's 110 profiled tumors. Rows mapping to a common symbol were summarized by their median. Protein features with more than 20% missing values were excluded; remaining missing values were median-imputed. In total, 9360 paired genes were retained.

Each gene was standardized across tumors separately within RNA and protein assays, and pathway scores were the mean standardized abundance of measured member genes. Among pathways eligible for the

prognostic study, assay covariance required at least 10 paired genes and 50% of original membership. All 38 qualifying pathways were tested. Spearman correlations used paired tumor measurements, 2000 paired bootstrap resamples for CIs and Benjamini–Hochberg correction across pathways (Table S10; Figure S1).

This representation describes interpatient RNA–protein covariance. It differs from the within-patient ranks of the survival model, uses no survival endpoint, and supplies no validation of the 16-gene prognostic predictor. No CPTAC result contributed to prognostic feature selection, model tuning or baseline estimation.

Supplementary Methods S8. Specification sensitivity

The original gene and pathway specifications were reproduced as numerical controls. Gene sensitivities changed one element at a time: L1 mixing fraction 0.1 or 0.9 instead of 0.5; within-training-fold variance-filter sizes 100 or 1000 instead of 500; or a 13-value penalty grid of 0.005, 0.0075, 0.01, 0.015, 0.025, 0.035, 0.05, 0.075, 0.10, 0.15, 0.25, 0.35 and 0.50. Pathway sensitivities used the same alternative mixing fractions or denser grid. Clinical terms remained unpenalized in molecular Cox models.

A lower-dimensional pathway model used the first five principal components of the 43 training-standardized pathway scores. Imputation, scaling and principal-component estimation were repeated within each source-held-out training fold. Clinical variables were standardized separately within the combined design matrix and kept unpenalized. A Breslow-tie Cox model used ridge penalties of 0.01, 0.1, 1, 10 or 100 on the five component coefficients. The component count was fixed at five; survival outcomes did not determine the component directions. Component variance explained is descriptive of expression variation, not prognostic information.

All configurations selected their penalty by the equal-source mean 1–3-year IBS using only the two development sources. Nonconvergent or numerically failed candidates were infeasible, following the original rule. Selected configurations were fitted to all 228 development patients and frozen before their external predictions were computed. All 11 configurations, including two original-specification controls, are reported in Tables S28–S29 and Figure S3. Original gene and pathway predictions were reproduced to an absolute difference below 10^{-12} in every external patient at all 25 evaluation times.

External comparisons used the original frozen clinical benchmark. Within each cohort, 2000 paired patient bootstrap resamples re-estimated censoring weights and generated percentile 95% intervals. The original resampling streams were reused, so original-specification control intervals agree with the primary analysis. Intervals condition on the fitted models and are unadjusted across these exploratory comparisons. Configurations with zero molecular coefficients can differ from the clinical reference by approximately 10^{-6} because the reference uses a small clinical ridge penalty whereas the molecular models leave clinical coefficients unpenalized. These small differences do not represent molecular information. The external results did not determine a replacement predictor.

Supplementary Tables

Tables use the same eligible populations and model definitions as the main text. Display values are rounded; full-precision calculations determine all contrasts and intervals. Reference numbers refer to the main manuscript bibliography.

Table S1. Endpoint provenance and harmonization.

Cohort	Event / time field	Units to years	Time origin support	Source location
GSE68465	vital_status; months_to_last_contact_or_death	Months / 12	Resection origin supported by published cohort analysis; individual dates unavailable	References [1,17]; [17] Materials and Methods, Statistical analysis, OS definition.
GSE72094	vital_status; survival_time_in_days	Days / 365.25	Resection origin in Moffitt investigators’ analysis	References [13,17]; [17] Statistical analysis, OS definition and MLOS cohort description.
GSE42127	survival status; overall survival months	Months / 12	Surgery explicitly identified in source study	Reference [14], Materials and Methods, statistical survival definition.
GSE50081	status; survival time	Years, unchanged	Surgery origin in published secondary analysis; original calendar dates unavailable	References [15,18]; [18] Materials and Methods, patient datasets paragraph and OS definition.
GSE31210	death; days before death/censor	Days / 365.25	Surgery origin in published secondary analysis; unit cross-check performed	References [16,18]; [18] patient datasets paragraph. Explicit days field agrees with duplicated OS-months field × 30.

The original event coding is converted to 1 for death and 0 for censored/alive. A published time-origin description is not a reconstruction of calendar dates. References are numbered in the main manuscript.

Table S2. Sequential eligibility accounting.

Cohort	Reason	n
Japan	included	226
Japan	not target histology or normal	20
MD Anderson	included	109
MD Anderson	invalid or missing survival	2
MD Anderson	not target histology or normal	43
MD Anderson	outside stage I II or stage missing	22
Toronto	included	127
Toronto	not target histology or normal	54
Development	development source overlap precaution	143
Development	included	228
Development	not target histology or normal	19
Development	outside stage I II or stage missing	72
Moffitt	included	316
Moffitt	invalid or missing survival	12
Moffitt	outside stage I II or stage missing	114

Table S3. Hallmark coverage on the known four-platform gene universe.

Pathway	Original genes	Measured genes	Coverage
ADIPOGENESIS	200	155	0.7750
ALLOGRAFT REJECTION	200	181	0.9050
ANDROGEN RESPONSE	101	86	0.8515
ANGIOGENESIS	36	34	0.9444
APICAL JUNCTION	200	160	0.8000
APICAL SURFACE	44	31	0.7045
APOPTOSIS	161	149	0.9255
BILE ACID METABOLISM	112	97	0.8661
CHOLESTEROL HOMEOSTASIS	74	57	0.7703
COAGULATION	138	130	0.9420
COMPLEMENT	200	173	0.8650
DNA REPAIR	150	120	0.8000
E2F TARGETS	200	157	0.7850
EPITHELIAL MESENCHYMAL TRANSITION	200	179	0.8950
ESTROGEN RESPONSE EARLY	200	171	0.8550
ESTROGEN RESPONSE LATE	200	183	0.9150
FATTY ACID METABOLISM	158	134	0.8481
G2M CHECKPOINT	200	164	0.8200
GLYCOLYSIS	200	172	0.8600
HEDGEHOG SIGNALING	36	34	0.9444
HEME METABOLISM	200	169	0.8450
HYPOXIA	200	175	0.8750
IL2 STAT5 SIGNALING	199	161	0.8090
IL6 JAK STAT3 SIGNALING	87	84	0.9655
INFLAMMATORY RESPONSE	200	184	0.9200
INTERFERON ALPHA RESPONSE	97	72	0.7423
INTERFERON GAMMA RESPONSE	200	166	0.8300
KRAS SIGNALING DN	200	174	0.8700
KRAS SIGNALING UP	200	180	0.9000
MITOTIC SPINDLE	199	162	0.8141
MTORC1 SIGNALING	200	173	0.8650
MYC TARGETS V1	200	160	0.8000
MYC TARGETS V2	58	45	0.7759
MYOGENESIS	200	188	0.9400
NOTCH SIGNALING	32	26	0.8125
OXIDATIVE PHOSPHORYLATION	200	166	0.8300
P53 PATHWAY	200	170	0.8500
PANCREAS BETA CELLS	40	36	0.9000
PEROXISOME	104	90	0.8654
PI3K AKT MTOR SIGNALING	105	95	0.9048
PROTEIN SECRETION	96	85	0.8854
REACTIVE OXYGEN SPECIES PATHWAY	49	38	0.7755
SPERMATOGENESIS	135	112	0.8296
TGF BETA SIGNALING	54	48	0.8889
TNFA SIGNALING VIA NFKB	200	178	0.8900
UNFOLDED PROTEIN RESPONSE	113	92	0.8142
UV RESPONSE DN	144	127	0.8819
UV RESPONSE UP	158	140	0.8861
WNT BETA CATENIN SIGNALING	42	35	0.8333
XENOBIOTIC METABOLISM	200	176	0.8800

Eligibility requires at least 10 measured genes and coverage ≥ 0.80. All 50 sets are shown.

Table S4. Prespecified model grids and selected final settings.

Model	Candidate settings	Selected
Clinical Cox	Ridge 0.01	0.01
Pathway Cox elastic net	α = 0.005, 0.015, 0.05, 0.15, 0.5; L1 fraction = 0.5	0.15; 0 molecular terms
Gene Cox elastic net	Same α grid; 500 training-variance genes	0.15; 16 molecular terms
Random survival forest	200 trees; leaf size 10 or 20; sqrt features	Leaf size 20
Gradient boosting	100 trees; depth/rate 1/0.03, 1/0.10, 2/0.03	1/0.03
ISRM gene-set refit	Same elastic-net grid; five published genes	0.15; 0 molecular terms

Settings were selected using development data only. Numerically failed or explicitly nonconvergent fits were infeasible candidates.

Table S5. Nested source-held-out performance and molecular selection.

Model	Held-out source	Test n	IBS	Uno C	3-year AUC	Molecular terms
Clinical	MI	145	0.1412	0.6020	0.6108	0
Clinical	MSKCC	83	0.0901	0.6036	0.6081	0
Pathway	MI	145	0.1412	0.6020	0.6108	0
Pathway	MSKCC	83	0.0901	0.6036	0.6081	0
Gene	MI	145	0.1412	0.6020	0.6108	0
Gene	MSKCC	83	0.0901	0.6036	0.6081	0
Forest	MI	145	0.1403	0.5343	0.5300	—
Forest	MSKCC	83	0.0964	0.3523	0.3834	—
Boosting	MI	145	0.1425	0.5289	0.5072	—
Boosting	MSKCC	83	0.0922	0.6469	0.6549	—
ISRM refit	MI	145	0.1412	0.6020	0.6108	—
ISRM refit	MSKCC	83	0.0901	0.6036	0.6081	—

MI denotes Michigan. The held-out MI fold trains on 83 MSKCC patients; the held-out MSKCC fold trains on 145 MI patients. Molecular counts are shown for the clinical, pathway and gene Cox fits. A dash indicates that the count is not tabulated for the other comparators.

Table S6. Complete frozen external performance.

Cohort	Model	n	IBS	Uno C	3-year AUC
Moffitt	Clinical	316	0.14095	0.61649	0.60676
Moffitt	Pathway	316	0.14095	0.61649	0.60676
Moffitt	Gene	316	0.13757	0.63004	0.63852
Moffitt	Forest	316	0.14651	0.60468	0.64670
Moffitt	Boosting	316	0.14747	0.49866	0.50560
Moffitt	ISRM refit	316	0.14095	0.61649	0.60676
MD Anderson	Clinical	109	0.08786	0.65137	0.63564
MD Anderson	Pathway	109	0.08786	0.65137	0.63564
MD Anderson	Gene	109	0.09071	0.66511	0.65529
MD Anderson	Forest	109	0.08575	0.61381	0.70549
MD Anderson	Boosting	109	0.08711	0.53458	0.53300
MD Anderson	ISRM refit	109	0.08786	0.65137	0.63564
Toronto	Clinical	127	0.13917	0.61118	0.63277
Toronto	Pathway	127	0.13917	0.61118	0.63277
Toronto	Gene	127	0.14469	0.60355	0.63188
Toronto	Forest	127	0.13990	0.64011	0.67573
Toronto	Boosting	127	0.14386	0.48763	0.48957
Toronto	ISRM refit	127	0.13917	0.61118	0.63277
Japan	Clinical	226	0.04905	0.79744	0.80729
Japan	Pathway	226	0.04905	0.79744	0.80729
Japan	Gene	226	0.05028	0.76076	0.77188
Japan	Forest	226	0.05428	0.56599	0.60164
Japan	Boosting	226	0.05346	0.72836	0.73732
Japan	ISRM refit	226	0.04905	0.79744	0.80729

IBS integrates 1–3 years. The pathway and ISRM refit retained no molecular coefficients; residual tiny differences from the clinical reference are numerical and clinical-penalty effects.

Table S7. Smoking-extended clinical comparison.

Cohort	Model	n	IBS	ΔIBS	95% lower	95% upper
Moffitt	Clinical	316	0.1409	0.0000	0.0000	0.0000
Moffitt	Pathway	316	0.1409	0.0000	0.0000	0.0000
Moffitt	Gene	316	0.1376	-0.0033	-0.0069	0.0004
Toronto	Clinical	127	0.1386	0.0000	0.0000	0.0000
Toronto	Pathway	127	0.1386	0.0000	0.0000	0.0000
Toronto	Gene	127	0.1442	0.0056	-0.0024	0.0128
Japan	Clinical	226	0.0483	0.0000	0.0000	0.0000
Japan	Pathway	226	0.0483	0.0000	0.0000	0.0000
Japan	Gene	226	0.0499	0.0017	-0.0006	0.0040

Each contrast evaluates models in the same patients. Stage I and original-eligibility comparisons retain the primary frozen models; smoking extension uses development-fitted smoking-extended models. Near-zero pathway differences reflect collapsed models. All comparisons are exploratory.

Table S8. Stage I and original prognosis-eligible comparisons.

Cohort	Population subset	Model	n	ΔIBS	95% lower	95% upper
Moffitt	Stage I	Pathway	249	0.0000	0.0000	0.0000
Moffitt	Stage I	Gene	249	-0.0022	-0.0047	0.0001
Moffitt	Stage I	Forest	249	0.0011	-0.0029	0.0053
Moffitt	Stage I	Boosting	249	0.0023	-0.0019	0.0064
MD Anderson	Stage I	Pathway	87	0.0000	0.0000	0.0000
MD Anderson	Stage I	Gene	87	0.0027	-0.0013	0.0064
MD Anderson	Stage I	Forest	87	0.0002	-0.0045	0.0047
MD Anderson	Stage I	Boosting	87	0.0014	-0.0036	0.0076
Toronto	Stage I	Pathway	92	0.0000	0.0000	0.0000
Toronto	Stage I	Gene	92	0.0051	-0.0022	0.0114
Toronto	Stage I	Forest	92	-0.0029	-0.0086	0.0031
Toronto	Stage I	Boosting	92	0.0004	-0.0054	0.0071
Japan	Stage I	Pathway	168	0.0000	0.0000	0.0000
Japan	Stage I	Gene	168	0.0008	-0.0003	0.0018
Japan	Stage I	Forest	168	0.0039	0.0028	0.0050
Japan	Stage I	Boosting	168	0.0025	0.0013	0.0037
Japan	Original eligible	Pathway	204	0.0000	0.0000	0.0000
Japan	Original eligible	Gene	204	0.0008	-0.0017	0.0030
Japan	Original eligible	Forest	204	0.0064	0.0014	0.0119
Japan	Original eligible	Boosting	204	0.0058	0.0005	0.0116

Each contrast evaluates models in the same patients. Stage I and original-eligibility comparisons retain the primary frozen models; smoking extension uses development-fitted smoking-extended models. Near-zero pathway differences reflect collapsed models. All comparisons are exploratory.

Table S9. Paired incremental error after cross-fitted baseline updating.

Population / summary	Updated clinical IBS	Updated gene IBS	Updated ΔIBS (95% CI)	Frozen ΔIBS
Moffitt	0.1408	0.1368	-0.0040 (-0.0079, 0.0003)	-0.0034
MD Anderson	0.0871	0.0862	-0.0009 (-0.0042, 0.0017)	0.0029
Toronto	0.1431	0.1449	0.0018 (-0.0012, 0.0054)	0.0055
Japan	0.0450	0.0449	-0.0001 (-0.0011, 0.0009)	0.0012
Equal cohort mean	0.1040	0.1032	-0.0008 (-0.0024, 0.0007)	0.0016
Sample size mean	0.1058	0.1043	-0.0015 (-0.0033, 0.0004)	0.0003

Both models use the same local five-fold partition. Each of 2000 bootstrap replicates resamples patients within their original folds, re-estimates both baseline hazards and recomputes censoring weights. Intervals condition on the frozen coefficients and original fold allocation. Negative differences favor the gene model. Summary absolute IBS values use the stated cohort weights.

Table S10. RNA–protein pathway covariance.

Pathway	Paired genes	Spearman ρ	95% lower	95% upper	BH q
ALLOGRAFT REJECTION	123	0.6054	0.4421	0.7388	7.56e-11
ANDROGEN RESPONSE	76	0.2535	0.0633	0.4398	0.01
ANGIOGENESIS	29	0.5665	0.3898	0.7084	1.56e-09
APICAL JUNCTION	139	0.4517	0.2711	0.6028	2.62e-06
APOPTOSIS	113	0.4076	0.2223	0.5707	2.29e-05
BILE ACID METABOLISM	71	0.4162	0.2361	0.5743	1.50e-05
COAGULATION	118	0.2997	0.0972	0.4771	0.0023
COMPLEMENT	152	0.4638	0.2808	0.6212	1.62e-06
DNA REPAIR	128	0.1880	0.0032	0.3687	0.057
EPITHELIAL MESENCHYMAL TRANSITION	159	0.7083	0.5547	0.8238	7.75e-16
ESTROGEN RESPONSE EARLY	127	0.4929	0.3330	0.6234	3.02e-07
ESTROGEN RESPONSE LATE	126	0.3615	0.1787	0.5299	0.0002
FATTY ACID METABOLISM	124	0.3643	0.1978	0.5308	0.00018
G2M CHECKPOINT	135	0.5377	0.3684	0.6662	1.33e-08
GLYCOLYSIS	150	0.4600	0.2632	0.6244	1.91e-06
HEDGEHOG SIGNALING	22	0.4582	0.2806	0.6061	1.99e-06
HEME METABOLISM	145	0.1910	0.0003	0.3794	0.054
HYPOXIA	140	0.4562	0.2625	0.6339	2.12e-06
IL2 STAT5 SIGNALING	118	0.4442	0.2647	0.6003	3.90e-06
IL6 JAK STAT3 SIGNALING	48	0.6030	0.4363	0.7353	7.56e-11
INTERFERON GAMMA RESPONSE	143	0.6758	0.5223	0.7980	3.64e-14
KRAS SIGNALING UP	116	0.4996	0.3103	0.6644	2.10e-07
MITOTIC SPINDLE	172	0.2122	0.0283	0.3904	0.032
MTORC1 SIGNALING	160	0.4348	0.2521	0.6065	5.80e-06
MYC TARGETS V1	188	0.4331	0.2391	0.5974	6.09e-06
MYOGENESIS	125	0.5678	0.4303	0.6794	1.56e-09
NOTCH SIGNALING	20	0.1659	-0.0445	0.3529	0.092
OXIDATIVE PHOSPHORYLATION	180	0.3332	0.1474	0.5027	0.00063
P53 PATHWAY	122	0.4432	0.2737	0.5890	3.92e-06
PEROXISOME	85	0.3922	0.2140	0.5549	4.87e-05
PI3K AKT MTOR SIGNALING	83	-0.0053	-0.2017	0.1945	0.96
PROTEIN SECRETION	91	0.2587	0.0744	0.4300	0.0088
TGF BETA SIGNALING	33	0.2859	0.0919	0.4449	0.0036
TNFA SIGNALING VIA NFKB	100	0.5523	0.3777	0.6860	4.53e-09
UNFOLDED PROTEIN RESPONSE	98	0.4805	0.2945	0.6386	6.43e-07
UV RESPONSE DN	113	0.4353	0.2462	0.5912	5.80e-06
UV RESPONSE UP	107	0.3495	0.1651	0.5127	0.00033
XENOBIOTIC METABOLISM	132	0.4642	0.2909	0.6104	1.62e-06

All 38 eligible pathways are shown. Each uses 106 matched tumors; intervals use 2000 paired tumor bootstrap resamples. q values adjust the pathway family.

Table S11. Clinical and nonzero molecular coefficients of the frozen gene model.

Feature	Coefficient	Training mean	Training SD
age	0.258205	64.793860	9.936048
male	0.243542	0.460526	0.498439
stage	0.288324	0.214912	0.410761
MUC5AC	0.108184	2925.750000	2629.018684
EREG	0.075208	2785.980263	2568.415824
RNF128	0.000518	5427.535088	2467.600771
HOXC10	-0.007009	4433.396930	2262.275277
PSPH	0.046230	3951.087719	2258.629619
NMNAT2	-0.048888	3723.427632	2163.110238
TNNC1	-0.041478	2065.572368	2120.751121
COL4A5	-0.077021	5490.734649	1942.185331
KCNQ1	-0.019060	3784.368421	1938.513351
CHST3	0.053911	3746.188596	1913.763733
TSPYL5	-0.007643	6958.188596	1858.267040
MAOA	-0.026162	5964.274123	1835.143171
FZD10	-0.008123	3238.462719	1821.029118
PITX1	0.018988	1937.605263	1820.560739
PLEK2	0.108383	5681.719298	1803.580595
CPM	-0.009290	6779.105263	1797.810276

Clinical features are age in years, male sex and broad stage II. Molecular features are within-patient gene ranks. Each feature is centered and scaled using the reported development mean and SD. The gene model contains 16 nonzero molecular terms.

Table S12. Clinical-only and pathway models within the same algorithm.

Cohort	Algorithm	Clinical IBS	Pathway IBS	ΔIBS	95% lower	95% upper
Moffitt	Forest	0.1422	0.1465	0.0043	-0.0028	0.0120
Moffitt	Boosting	0.1429	0.1475	0.0046	-0.0035	0.0130
MD Anderson	Forest	0.0846	0.0857	0.0011	-0.0075	0.0105
MD Anderson	Boosting	0.0863	0.0871	0.0008	-0.0066	0.0095
Toronto	Forest	0.1421	0.1399	-0.0022	-0.0116	0.0077
Toronto	Boosting	0.1389	0.1439	0.0050	-0.0082	0.0181
Japan	Forest	0.0471	0.0543	0.0072	0.0025	0.0127
Japan	Boosting	0.0503	0.0535	0.0031	-0.0010	0.0080

These exploratory controls use the same candidate grids and development-only source tuning. Negative contrasts favor added pathway predictors.

Table S13. Censoring tie convention.

Cohort	Model	Primary IBS	Event-first IBS	Difference
Moffitt	Clinical	0.140950	0.140961	0.000011
Moffitt	Pathway	0.140950	0.140961	0.000011
Moffitt	Gene	0.137573	0.137583	0.000011
Moffitt	Forest	0.146512	0.146522	0.000010
Moffitt	Boosting	0.147468	0.147478	0.000010
Moffitt	ISRM refit	0.140950	0.140961	0.000011
MD Anderson	Clinical	0.087857	0.087882	0.000025
MD Anderson	Pathway	0.087858	0.087883	0.000025
MD Anderson	Gene	0.090708	0.090738	0.000029
MD Anderson	Forest	0.085747	0.085768	0.000021
MD Anderson	Boosting	0.087109	0.087129	0.000020
MD Anderson	ISRM refit	0.087858	0.087883	0.000025
Toronto	Clinical	0.139168	0.139168	0.000000
Toronto	Pathway	0.139168	0.139168	0.000000
Toronto	Gene	0.144688	0.144688	0.000000
Toronto	Forest	0.139902	0.139902	0.000000
Toronto	Boosting	0.143864	0.143864	0.000000
Toronto	ISRM refit	0.139168	0.139168	0.000000
Japan	Clinical	0.049053	0.049053	0.000000
Japan	Pathway	0.049052	0.049052	0.000000
Japan	Gene	0.050285	0.050285	0.000000
Japan	Forest	0.054280	0.054280	0.000000
Japan	Boosting	0.053460	0.053460	0.000000
Japan	ISRM refit	0.049052	0.049052	0.000000

The event-first convention removes tied deaths before estimating the censoring risk denominator. Differences are numerical sensitivity to this convention, not a test of independent censoring.

Table S14. Three-year calibration-group support and transported cutpoints.

Cohort	Model	Group	n	Deaths by 3 y	At risk at 3 y	Lower risk	Upper risk
Moffitt	Clinical	1	91	14	17	0.000	0.162
Moffitt	Clinical	2	83	14	20	0.162	0.222
Moffitt	Clinical	3	142	41	23	0.222	1.000
MD Anderson	Clinical	1	45	4	29	0.000	0.162
MD Anderson	Clinical	2	24	3	17	0.162	0.222
MD Anderson	Clinical	3	40	11	24	0.222	1.000
Toronto	Clinical	1	34	6	24	0.000	0.162
Toronto	Clinical	2	27	4	20	0.162	0.222
Toronto	Clinical	3	66	19	40	0.222	1.000
Japan	Clinical	1	102	3	96	0.000	0.162
Japan	Clinical	2	62	4	50	0.162	0.222
Japan	Clinical	3	62	13	39	0.222	1.000
Moffitt	Gene	1	98	16	21	0.000	0.153
Moffitt	Gene	2	106	14	22	0.153	0.231
Moffitt	Gene	3	112	39	17	0.231	1.000
MD Anderson	Gene	1	30	2	19	0.000	0.153
MD Anderson	Gene	2	29	4	19	0.153	0.231
MD Anderson	Gene	3	50	12	32	0.231	1.000
Toronto	Gene	1	15	4	11	0.000	0.153
Toronto	Gene	2	27	3	19	0.153	0.231
Toronto	Gene	3	85	22	54	0.231	1.000
Japan	Gene	1	93	3	86	0.000	0.153
Japan	Gene	2	67	4	56	0.153	0.231
Japan	Gene	3	66	13	43	0.231	1.000

Risk-tertile cutpoints were derived in development separately by model and applied unchanged to each cohort.

Table S15. Development-bootstrap selection summary.

Quantity	Value
Requested / completed resamples	200 / 200
Resamples retaining no molecular terms	159 (79.5%)
Resamples retaining ≥1 molecular term	41 (20.5%)
Nonzero count: median (25th, 75th percentile)	0 (0, 0)
Nonzero count: range	0–95
Selected penalty 0.50	160 (80.0%)
Selected penalty 0.15	38 (19.0%)
Selected penalty 0.05	2 (1.0%)
Selected penalty 0.005 or 0.015	0
Final-gene reselection frequency: range	3.5%–16.5%

Each draw preserves the two development-source sizes and repeats feature filtering, source-separated tuning and fitting. A selected penalty of 0.50 retained one molecular coefficient in one resample; penalty choice and model collapse are therefore distinct quantities.

Table S16. Gene reselection frequencies.

Gene	Selected / 200	Frequency (%)	In final 16-gene model
MUC5AC	33	16.5	Yes
EREG	32	16.0	Yes
PLEK2	29	14.5	Yes
COL4A5	25	12.5	Yes
NMNAT2	23	11.5	Yes
MAOA	22	11.0	Yes
PSPH	20	10.0	Yes
CPM	20	10.0	Yes
TNNC1	19	9.5	Yes
CHST3	14	7.0	Yes
KCNQ1	13	6.5	Yes
FZD10	13	6.5	Yes
PITX1	10	5.0	Yes
HOXC10	10	5.0	Yes
TSPYL5	10	5.0	Yes
RNF128	7	3.5	Yes

All final-model genes and any additional gene selected in at least 10% of resamples are shown. The denominator includes collapsed fits; frequencies reflect screening and penalized selection together.

Table S17. Correlation with the frozen gene-model prognostic index.

Cohort	Total n	Total ρ: median (IQR)	Molecular n	Molecular ρ: median (IQR)
Development	200	0.819 (0.783, 0.838)	41	0.653 (0.568, 0.703)
Moffitt	200	0.901 (0.840, 0.913)	41	0.584 (0.483, 0.670)
MD Anderson	200	0.922 (0.884, 0.928)	41	0.559 (0.515, 0.666)
Toronto	200	0.921 (0.850, 0.930)	41	0.557 (0.428, 0.636)
Japan	200	0.909 (0.867, 0.920)	41	0.635 (0.564, 0.721)

Total-index summaries use all 200 completed fits. Molecular-only summaries use the 41 noncollapsed fits. IQR denotes the 25th and 75th percentiles of bootstrap index correlations, not a confidence interval.

Table S18. Complementary cohort aggregation.

Model	Weights	ΔIBS (95% CI)	Relative change, % (95% CI)
Gene	Equal cohort	0.0016 (-0.0012, 0.0042)	1.83 (-0.70, 5.32)
Gene	Sample size	0.0003 (-0.0019, 0.0025)	0.86 (-1.31, 3.35)
Forest	Equal cohort	0.0024 (-0.0015, 0.0065)	3.18 (-1.32, 6.96)
Forest	Sample size	0.0036 (-0.0002, 0.0075)	4.45 (0.40, 8.07)
Boosting	Equal cohort	0.0037 (-0.0005, 0.0081)	4.03 (-1.07, 8.19)
Boosting	Sample size	0.0046 (0.0008, 0.0087)	4.92 (0.58, 8.77)

Equal weighting targets the mean effect across four specific populations; sample weighting uses n/778. Relative error change is calculated within each cohort before weighting.

Table S18 (continued). Descriptive between-cohort heterogeneity.

Model	Q	df	p	I^2 (%)
Gene	7.031	3	0.071	57.331
Forest	2.332	3	0.506	0.000
Boosting	1.206	3	0.752	0.000

Within-cohort variances come from the conditional frozen-model bootstrap. Four cohorts provide limited information about heterogeneity.

Table S19. Censoring-weight support across horizons.

Cohort	Year	G(t)	Nonzero n	Median w	95th pct w	Max w	ESS
Moffitt	1.000	0.938	297	1.067	1.067	1.067	296.975
Moffitt	2.000	0.781	255	1.280	1.280	1.280	253.782
Moffitt	2.500	0.492	182	2.031	2.031	2.031	171.314
Moffitt	3.000	0.266	129	2.345	3.757	3.757	101.534
MD Anderson	1.000	0.972	106	1.028	1.028	1.028	106.000
MD Anderson	2.000	0.874	96	1.144	1.144	1.144	95.935
MD Anderson	2.500	0.822	91	1.216	1.216	1.216	90.835
MD Anderson	3.000	0.790	88	1.266	1.266	1.266	87.735
Toronto	1.000	0.952	121	1.050	1.050	1.050	120.998
Toronto	2.000	0.907	116	1.103	1.103	1.103	115.959
Toronto	2.500	0.907	116	1.103	1.103	1.103	115.959
Toronto	3.000	0.876	113	1.141	1.141	1.141	112.894
Japan	1.000	1.000	226	1.000	1.000	1.000	226.000
Japan	2.000	0.981	222	1.019	1.019	1.019	221.997
Japan	2.500	0.930	211	1.076	1.076	1.076	210.946
Japan	3.000	0.901	205	1.110	1.110	1.110	204.885

Quantiles describe nonzero IPCW contributions. ESS = (sum of weights)^2 / sum of squared weights. Risk-set counts are distinct from nonzero outcome contributions; Moffitt has 60 patients at risk, 129 nonzero contributions and ESS 101.5 at 3 years.

Table S20. Follow-up horizon and capped-weight sensitivity.

Cohort	Horizon, y	Weight cap	Clinical IBS	Gene IBS	ΔIBS (95% CI)
Moffitt	2.0000	None	0.1114	0.1090	-0.0024 (-0.0046, -0.0003)
Moffitt	2.5000	None	0.1252	0.1222	-0.0031 (-0.0060, -0.0003)
Moffitt	3.0000	None	0.1409	0.1376	-0.0034 (-0.0070, 0.0002)
Moffitt	3.0000	3	0.1406	0.1373	-0.0033 (-0.0069, 0.0002)
Moffitt	3.0000	5	0.1409	0.1376	-0.0034 (-0.0070, 0.0002)
Moffitt	3.0000	10	0.1409	0.1376	-0.0034 (-0.0070, 0.0002)
MD Anderson	2.0000	None	0.0591	0.0600	0.0009 (-0.0041, 0.0052)
MD Anderson	2.5000	None	0.0728	0.0750	0.0022 (-0.0038, 0.0075)
MD Anderson	3.0000	None	0.0879	0.0907	0.0029 (-0.0038, 0.0091)
MD Anderson	3.0000	3	0.0879	0.0907	0.0029 (-0.0038, 0.0091)
MD Anderson	3.0000	5	0.0879	0.0907	0.0029 (-0.0038, 0.0091)
MD Anderson	3.0000	10	0.0879	0.0907	0.0029 (-0.0038, 0.0091)
Toronto	2.0000	None	0.1158	0.1195	0.0037 (-0.0018, 0.0092)
Toronto	2.5000	None	0.1308	0.1355	0.0047 (-0.0021, 0.0114)
Toronto	3.0000	None	0.1392	0.1447	0.0055 (-0.0022, 0.0130)
Toronto	3.0000	3	0.1392	0.1447	0.0055 (-0.0022, 0.0130)
Toronto	3.0000	5	0.1392	0.1447	0.0055 (-0.0022, 0.0130)
Toronto	3.0000	10	0.1392	0.1447	0.0055 (-0.0022, 0.0130)
Japan	2.0000	None	0.0340	0.0345	0.0004 (-0.0009, 0.0017)
Japan	2.5000	None	0.0416	0.0424	0.0008 (-0.0012, 0.0026)
Japan	3.0000	None	0.0491	0.0503	0.0012 (-0.0011, 0.0035)
Japan	3.0000	3	0.0491	0.0503	0.0012 (-0.0011, 0.0035)
Japan	3.0000	5	0.0491	0.0503	0.0012 (-0.0011, 0.0035)
Japan	3.0000	10	0.0491	0.0503	0.0012 (-0.0011, 0.0035)

Each interval uses 2000 paired resamples with censoring weights recomputed. All integrations start at year 1. The same resampling stream as the primary analysis ensures agreement of the uncapped 1–3-year estimate and interval.

Table S21. Calibration at 1, 2 and 3 years.

Cohort	Model	Year	Predicted mortality, %	Observed mortality, % (95% CI)	Slope (95% CI)
Development	Clinical	1.0	6.2	6.2 (3.7, 10.2)	1.77 (0.60, 2.95)
Development	Clinical	2.0	16.6	16.4 (12.2, 21.9)	1.45 (0.70, 2.20)
Development	Clinical	3.0	20.8	20.6 (15.8, 26.5)	1.29 (0.62, 1.96)
Development	Gene	1.0	6.3	6.2 (3.7, 10.2)	2.21 (1.21, 3.20)
Development	Gene	2.0	16.9	16.4 (12.2, 21.9)	2.03 (1.38, 2.67)
Development	Gene	3.0	21.3	20.6 (15.8, 26.5)	1.87 (1.29, 2.46)
Moffitt	Clinical	1.0	6.8	9.5 (6.7, 13.4)	1.05 (0.29, 1.80)
Moffitt	Clinical	2.0	18.0	18.1 (14.2, 23.1)	0.88 (0.32, 1.44)
Moffitt	Clinical	3.0	22.6	28.6 (22.8, 35.5)	0.85 (0.36, 1.35)
Moffitt	Gene	1.0	6.4	9.5 (6.7, 13.4)	1.25 (0.59, 1.91)
Moffitt	Gene	2.0	17.2	18.1 (14.2, 23.1)	1.04 (0.55, 1.54)
Moffitt	Gene	3.0	21.7	28.6 (22.8, 35.5)	0.96 (0.53, 1.40)
MD Anderson	Clinical	1.0	6.3	3.8 (1.4, 9.7)	2.61 (0.48, 4.74)
MD Anderson	Clinical	2.0	16.8	8.7 (4.6, 16.0)	1.27 (0.02, 2.51)
MD Anderson	Clinical	3.0	21.0	18.6 (12.1, 28.0)	0.93 (0.06, 1.80)
MD Anderson	Gene	1.0	7.3	3.8 (1.4, 9.7)	3.05 (0.66, 5.44)
MD Anderson	Gene	2.0	19.5	8.7 (4.6, 16.0)	1.42 (0.25, 2.60)
MD Anderson	Gene	3.0	24.4	18.6 (12.1, 28.0)	0.97 (0.18, 1.77)
Toronto	Clinical	1.0	7.2	8.2 (4.5, 14.7)	0.22 (-1.10, 1.54)
Toronto	Clinical	2.0	19.1	19.2 (13.2, 27.5)	0.40 (-0.47, 1.26)
Toronto	Clinical	3.0	23.8	24.5 (17.7, 33.3)	0.88 (0.11, 1.66)
Toronto	Gene	1.0	9.5	8.2 (4.5, 14.7)	0.13 (-1.02, 1.27)
Toronto	Gene	2.0	24.8	19.2 (13.2, 27.5)	0.23 (-0.53, 0.98)
Toronto	Gene	3.0	30.7	24.5 (17.7, 33.3)	0.71 (0.03, 1.39)
Japan	Clinical	1.0	5.8	1.8 (0.7, 4.6)	4.15 (0.79, 7.52)
Japan	Clinical	2.0	15.6	5.3 (3.1, 9.2)	4.14 (2.26, 6.02)
Japan	Clinical	3.0	19.6	9.1 (6.0, 13.8)	2.46 (1.45, 3.48)
Japan	Gene	1.0	5.9	1.8 (0.7, 4.6)	3.26 (0.85, 5.67)
Japan	Gene	2.0	16.0	5.3 (3.1, 9.2)	2.90 (1.65, 4.14)
Japan	Gene	3.0	20.1	9.1 (6.0, 13.8)	1.76 (0.97, 2.56)

Observed mortality uses Kaplan–Meier estimation. Slopes use a local Cox model containing the frozen horizon-specific log cumulative hazard, with follow-up truncated at that horizon. Their CIs are model-based.

Table S22. Proportional-hazards diagnostics through 3 years.

Cohort	Model	Diagnostic	χ^2	df	p	BH q
Development	Clinical	Frozen index	1.3616	1	0.2433	0.55
Development	Clinical	Joint covariates	2.6795	3	0.4437	0.59
Development	Gene	Frozen index	0.8906	1	0.3453	0.58
Development	Gene	Joint covariates	1.7640	4	0.7791	0.82
Moffitt	Clinical	Frozen index	0.0091	1	0.9241	0.92
Moffitt	Clinical	Joint covariates	3.8904	3	0.2735	0.55
Moffitt	Gene	Frozen index	0.1324	1	0.7160	0.8
Moffitt	Gene	Joint covariates	4.2151	4	0.3777	0.58
MD Anderson	Clinical	Frozen index	2.6657	1	0.1025	0.34
MD Anderson	Clinical	Joint covariates	4.0590	3	0.2552	0.55
MD Anderson	Gene	Frozen index	3.8932	1	0.0485	0.27
MD Anderson	Gene	Joint covariates	5.9982	4	0.1993	0.55
Toronto	Clinical	Frozen index	0.6886	1	0.4067	0.58
Toronto	Clinical	Joint covariates	1.8893	3	0.5957	0.74
Toronto	Gene	Frozen index	0.8567	1	0.3547	0.58
Toronto	Gene	Joint covariates	2.2921	4	0.6822	0.8
Japan	Clinical	Frozen index	6.2121	1	0.0127	0.13
Japan	Clinical	Joint covariates	7.1753	3	0.0665	0.27
Japan	Gene	Frozen index	6.6346	1	0.0100	0.13
Japan	Gene	Joint covariates	8.8358	4	0.0653	0.27

Global efficient score tests assess centered log-event-time interactions. Joint covariates comprise age, sex and stage, plus the frozen molecular index for the gene model. BH adjustment covers 20 tests. No prediction model was changed using these diagnostics.

Table S23. Nonlinear-age diagnostics through 3 years.

Cohort	Model	Likelihood-ratio χ^2	df	p	BH q
Development	Clinical	3.5602	1	0.0592	0.42
Development	Gene	2.9801	1	0.0843	0.42
Moffitt	Clinical	0.9957	1	0.3184	0.8
Moffitt	Gene	1.2842	1	0.2571	0.8
MD Anderson	Clinical	0.0674	1	0.7951	0.99
MD Anderson	Gene	0.0161	1	0.8991	0.99
Toronto	Clinical	0.2459	1	0.6200	0.99
Toronto	Gene	0.2458	1	0.6201	0.99
Japan	Clinical	0.0110	1	0.9164	0.99
Japan	Gene	0.0003	1	0.9854	0.99

The additional restricted-cubic-spline term uses development knots 51, 65 and 77 years. The gene diagnostic additionally adjusts for the frozen molecular index. BH adjustment covers 10 tests.

Table S24. Clinical coefficients and centering offsets of the frozen Cox models.

Model	Age coefficient	Male coefficient	Stage II coefficient	Centering offset
Clinical	0.198531849	0.241509437	0.303872417	0.000000000e+00
Pathway	0.198570636	0.241558845	0.303918998	-2.679102060e-17
Gene	0.258204971	0.243541977	0.288323628	-4.601439146e-17
ISRM refit	0.198570636	0.241558845	0.303918998	-2.679102060e-17

Age is measured in years; male sex and stage II are coded 1, with female sex and stage I coded 0. Coefficients apply after training standardization. The training means and standard deviations for these three variables are identical across models and are reported in Table S11. Training imputation medians are 65 years for age, 0 for male sex and 0 for stage II. The gene model also uses the 16 nonzero molecular terms in Table S11. The pathway and ISRM refit models retain zero molecular coefficients. The centering offset is subtracted from the sum of standardized covariate contributions, as specified in Supplementary Methods S2.

Table S25. Frozen baseline survival functions through 3 years.

Time, years	Clinical S0(t)	Pathway S0(t)	Gene S0(t)	ISRM refit S0(t)
0.186666666667	0.996046059	0.996046204	0.996227331	0.996046204
0.275000000000	0.992096559	0.992096849	0.992457720	0.992096849
0.333333333333	0.988115958	0.988116386	0.988628951	0.988116386
0.483333333333	0.984135953	0.984136520	0.984792723	0.984136520
0.531666666667	0.980161439	0.980162145	0.980960857	0.980162145
0.550000000000	0.976154061	0.976154899	0.977101660	0.976154899
0.608333333333	0.972093047	0.972094009	0.973196130	0.972094009
0.700000000000	0.967997430	0.967998507	0.969268393	0.967998507
0.725000000000	0.963874321	0.963875509	0.965281878	0.963875509
0.737500000000	0.959760225	0.959761523	0.961302568	0.959761523
0.766666666667	0.955644234	0.955645643	0.957304381	0.955645643
0.833333333333	0.951525029	0.951526549	0.953288627	0.951526549
0.841666666667	0.947389725	0.947391353	0.949249514	0.947391353
0.973333333333	0.943249601	0.943251339	0.945193273	0.943251339
1.016666666667	0.939106608	0.939108456	0.941126033	0.939108456
1.041666666667	0.934959421	0.934961377	0.937046099	0.934961377
1.083333333333	0.930784921	0.930786980	0.932937905	0.930786980
1.166666666667	0.926612173	0.926614335	0.928829426	0.926614335
1.250000000000	0.918292188	0.918294556	0.920625013	0.918294556
1.416666666667	0.914108159	0.914110631	0.916478463	0.914110631
1.550000000000	0.909891689	0.909894258	0.912292667	0.909894258
1.583333333333	0.901466399	0.901469159	0.903896300	0.901469159
1.625000000000	0.897235656	0.897238510	0.899659623	0.897238510
1.658333333333	0.892996641	0.892999587	0.895403811	0.892999587
1.666666666667	0.884523252	0.884526377	0.886890994	0.884526377
1.716666666667	0.880241294	0.880244504	0.882545259	0.880244504
1.741666666667	0.875966441	0.875969735	0.878204021	0.875969735
1.750000000000	0.871691133	0.871694510	0.873866071	0.871694510
1.766666666667	0.867397480	0.867400939	0.869495597	0.867400939
1.790000000000	0.863105032	0.863108571	0.865128309	0.863108571
1.858333333333	0.858748681	0.858752300	0.860717422	0.858752300

Time, years	Clinical S0(t)	Pathway S0(t)	Gene S0(t)	ISRM refit S0(t)
1.891666666667	0.854385704	0.854389402	0.856292984	0.854389402
1.916666666667	0.850020334	0.850024110	0.851866961	0.850024110
2.000000000000	0.845654359	0.845658214	0.847430949	0.845658214
2.162500000000	0.841280746	0.841284683	0.842987431	0.841284683
2.300000000000	0.836834381	0.836838395	0.838473194	0.836838395
2.416666666667	0.832387858	0.832391950	0.833954566	0.832391950
2.516666666667	0.827888645	0.827892816	0.829344146	0.827892816
2.558333333333	0.823390233	0.823394483	0.824725880	0.823394483
2.661666666667	0.818866741	0.818871069	0.820090954	0.818871069
2.666666666667	0.814348538	0.814352945	0.815462567	0.814352945
2.825000000000	0.809618312	0.809622788	0.810655470	0.809622788
2.900000000000	0.804898899	0.804903445	0.805847052	0.804903445

Each row gives the baseline survival immediately after an event-time jump. Values remain constant until the next listed time; survival is 1 before the first jump, and the last listed value applies through 3 years. These are the frozen development baseline functions. Locally updated baseline functions are estimated within the evaluation folds as described in Supplementary Methods S5.

Table S26. Joint distribution of selected penalty and retained gene counts across 200 development resamples.

Penalty	Selected n (%)	Zero genes, n	Nonzero genes, n	Median genes (range)
0.005	0 (0.0)	0	0	—
0.015	0 (0.0)	0	0	—
0.050	2 (1.0)	0	2	89.5 (84–95)
0.150	38 (19.0)	0	38	29.0 (19–43)
0.500	160 (80.0)	159	1	0.0 (0–1)

Each resample repeated source-stratified patient sampling, variance filtering, penalty selection and fitting. Counts refer to nonzero molecular coefficients. The two smallest penalties were never selected. The complete retained-count distribution follows.

Table S26 (continued). Exact retained-gene count frequencies.

Retained genes	Resamples, n	Resamples, %
0	159	79.5
1	1	0.5
19	1	0.5
21	1	0.5
24	3	1.5
26	5	2.5
27	4	2.0
28	2	1.0
29	5	2.5
30	2	1.0
31	2	1.0
32	3	1.5
33	2	1.0
34	3	1.5
35	3	1.5
36	1	0.5
43	1	0.5
84	1	0.5
95	1	0.5

The frequency distribution includes all 200 completed resamples; its median and interquartile range are both zero.

Table S27. Original gene-model tuning scores for final source-based selection.

Penalty	Held-out Michigan IBS	Held-out MSKCC IBS	Equal-source mean IBS
0.005	Infeasible	0.228706	Infeasible
0.015	Infeasible	Infeasible	Infeasible
0.050	0.164342	0.138501	0.151421
0.150	0.140579	0.089753	0.115166
0.500	0.141222	0.090067	0.115645

For each candidate, preprocessing and variance filtering were fitted only in the other source. Infeasible denotes numerical failure or nonconvergence in at least one training fit. The minimum finite score selected penalty 0.15. Nested outer-fold fits used three-fold tuning inside the remaining source and selected penalty 0.5 (Table S5); their tuning problem was different from this final two-source selection.

Table S28. Development-only selection for alternative modeling specifications.

Specification	Mixing fraction	Candidate molecular terms	Penalty values, n	Selected penalty	Mean source IBS	Retained terms
Gene original	0.5	500	5	0.150	0.115166	16
Gene mixing 0.1	0.1	500	5	0.500	0.115901	61
Gene mixing 0.9	0.9	500	5	0.150	0.114510	1
Gene top 100	0.5	100	5	0.150	0.115154	7
Gene top 1000	0.5	1000	5	0.500	0.115848	0
Gene dense grid	0.5	500	13	0.250	0.114423	1
Pathway original	0.5	43	5	0.150	0.114359	0
Pathway mixing 0.1	0.1	43	5	0.500	0.114651	4
Pathway mixing 0.9	0.9	43	5	0.050	0.115348	5
Pathway dense grid	0.5	43	13	0.150	0.114359	0
Pathway five PCs	0.0	5	5	100.000	0.117009	5

Each alternative changes the stated feature of the original specification. Principal components (PCs) replace pathway scores; their directions were fitted without outcomes within training data, and five PCs explained 69.5% of full-development standardized pathway variance. The component ridge penalty has a different scaling convention from the elastic-net penalty and is not numerically comparable. The full grids are given in Supplementary Methods S8. All configurations were frozen before external prediction and none replaced the original model.

Table S29. Cohort-specific external performance of all assessed specifications.

Cohort	Specification	IBS	ΔIBS (95% CI)
Moffitt	Gene original	0.13757	-0.00338 (-0.00698, 0.00016)
Moffitt	Gene mixing 0.1	0.14071	-0.00024 (-0.00459, 0.00424)
Moffitt	Gene mixing 0.9	0.14081	-0.00014 (-0.00030, 1.38e-05)
Moffitt	Gene top 100	0.13880	-0.00215 (-0.00399, -0.00047)
Moffitt	Gene top 1000	0.14095	1.44e-07 (-1.24e-06, 1.53e-06)
Moffitt	Gene dense grid	0.14059	-0.00036 (-0.00079, 4.69e-05)
Moffitt	Pathway original	0.14095	1.44e-07 (-1.24e-06, 1.53e-06)
Moffitt	Pathway mixing 0.1	0.14052	-0.00043 (-0.00105, 0.00013)
Moffitt	Pathway mixing 0.9	0.13986	-0.00109 (-0.00279, 0.00049)
Moffitt	Pathway dense grid	0.14095	1.44e-07 (-1.24e-06, 1.53e-06)
Moffitt	Pathway five PCs	0.14209	0.00114 (-0.00064, 0.00305)
MD Anderson	Gene original	0.09071	0.00285 (-0.00384, 0.00908)
MD Anderson	Gene mixing 0.1	0.08968	0.00183 (-0.00477, 0.00851)
MD Anderson	Gene mixing 0.9	0.08782	-3.45e-05 (-0.00021, 0.00012)
MD Anderson	Gene top 100	0.08854	0.00069 (-0.00254, 0.00382)
MD Anderson	Gene top 1000	0.08786	9.32e-07 (-1.11e-06, 3.02e-06)
MD Anderson	Gene dense grid	0.08777	-0.00009 (-0.00057, 0.00034)
MD Anderson	Pathway original	0.08786	9.32e-07 (-1.11e-06, 3.02e-06)
MD Anderson	Pathway mixing 0.1	0.08657	-0.00129 (-0.00244, -0.00011)
MD Anderson	Pathway mixing 0.9	0.08513	-0.00273 (-0.00576, 0.00039)
MD Anderson	Pathway dense grid	0.08786	9.32e-07 (-1.11e-06, 3.02e-06)
MD Anderson	Pathway five PCs	0.08690	-0.00096 (-0.00490, 0.00366)
Toronto	Gene original	0.14469	0.00552 (-0.00222, 0.01299)
Toronto	Gene mixing 0.1	0.14145	0.00228 (-0.00368, 0.00806)
Toronto	Gene mixing 0.9	0.13919	2.13e-05 (-0.00031, 0.00032)
Toronto	Gene top 100	0.13885	-0.00031 (-0.00228, 0.00151)
Toronto	Gene top 1000	0.13917	7.27e-07 (-1.08e-06, 2.61e-06)
Toronto	Gene dense grid	0.13925	0.00008 (-0.00080, 0.00088)
Toronto	Pathway original	0.13917	7.27e-07 (-1.08e-06, 2.61e-06)
Toronto	Pathway mixing 0.1	0.14008	0.00091 (-0.00133, 0.00346)
Toronto	Pathway mixing 0.9	0.14189	0.00272 (-0.00234, 0.00841)
Toronto	Pathway dense grid	0.13917	7.27e-07 (-1.08e-06, 2.61e-06)
Toronto	Pathway five PCs	0.14346	0.00429 (-0.00346, 0.01255)
Japan	Gene original	0.05028	0.00123 (-0.00109, 0.00346)
Japan	Gene mixing 0.1	0.04979	0.00074 (-0.00239, 0.00370)
Japan	Gene mixing 0.9	0.04916	0.00010 (-9.27e-07, 0.00020)
Japan	Gene top 100	0.04989	0.00084 (-0.00043, 0.00211)
Japan	Gene top 1000	0.04905	-1.03e-06 (-1.98e-06, -1.35e-07)
Japan	Gene dense grid	0.04934	0.00029 (6.83e-06, 0.00054)
Japan	Pathway original	0.04905	-1.03e-06 (-1.98e-06, -1.35e-07)
Japan	Pathway mixing 0.1	0.04923	0.00018 (-0.00022, 0.00054)
Japan	Pathway mixing 0.9	0.04918	0.00013 (-0.00084, 0.00101)
Japan	Pathway dense grid	0.04905	-1.03e-06 (-1.98e-06, -1.35e-07)
Japan	Pathway five PCs	0.04753	-0.00152 (-0.00262, -0.00038)

Contrasts compare each molecular configuration with the same original frozen clinical model. Intervals use 2000 paired patient-bootstrap resamples with censoring weights re-estimated and condition on each fitted model. All are post hoc, multiplicity-unadjusted comparisons. Original-specification controls reproduce the primary results. Differences of approximately 10^{-6} for collapsed models arise from clinical-penalty and numerical differences; they are not molecular effects.

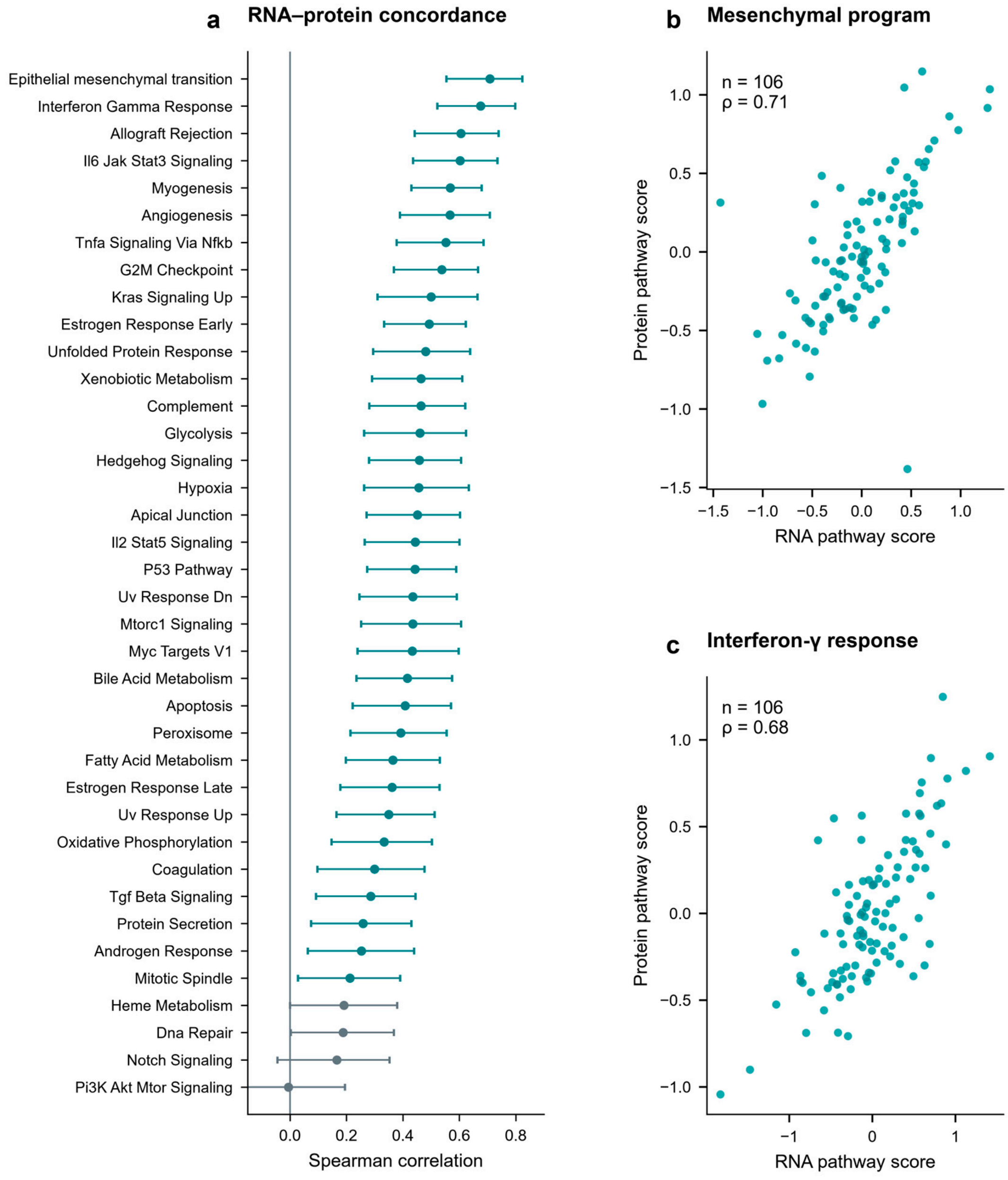

Figure S1. RNA–protein pathway concordance in 106 public CPTAC tumors. (a) Spearman correlations between matched RNA and protein pathway scores for all 38 evaluable Hallmark programs. Points are correlations; bars show 95% intervals from 2000 paired tumor bootstrap resamples. Teal indicates a positive correlation with Benjamini–Hochberg false discovery rate below 0.05; gray indicates the remaining programs. (b,c) Illustrative matched-tumor scores for epithelial–mesenchymal transition and interferon-γ response. Each point is one tumor. Scores are means of gene-wise standardized abundance within each assay and differ from the prognostic rank scores. This analysis evaluates biological covariance, not survival-model validation.

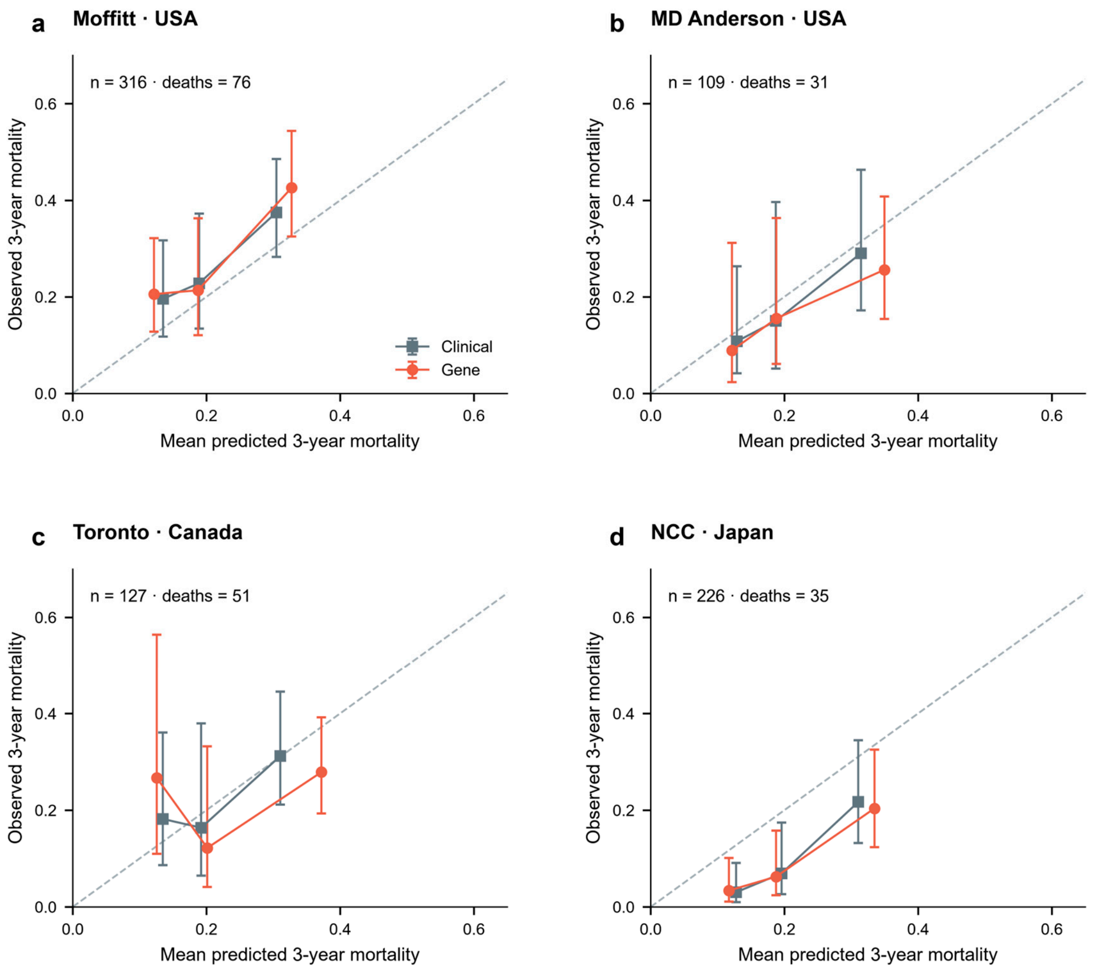

Figure S2. Calibration of frozen clinical and gene models at 3 years. Panels show (a) Moffitt, (b) MD Anderson, (c) Toronto and (d) the Japanese National Cancer Center cohort. Patients were grouped using risk-tertile cutpoints fixed in development. Points compare mean predicted mortality with Kaplan–Meier observed mortality; vertical bars show 95% Kaplan–Meier intervals. The dashed diagonal indicates perfect calibration. Lines connect groups for visual guidance and are not fitted calibration functions. Model-specific group counts, three-year support and cutpoints are supplied in Supplementary Table S14. Death counts in each panel refer to all available follow-up.

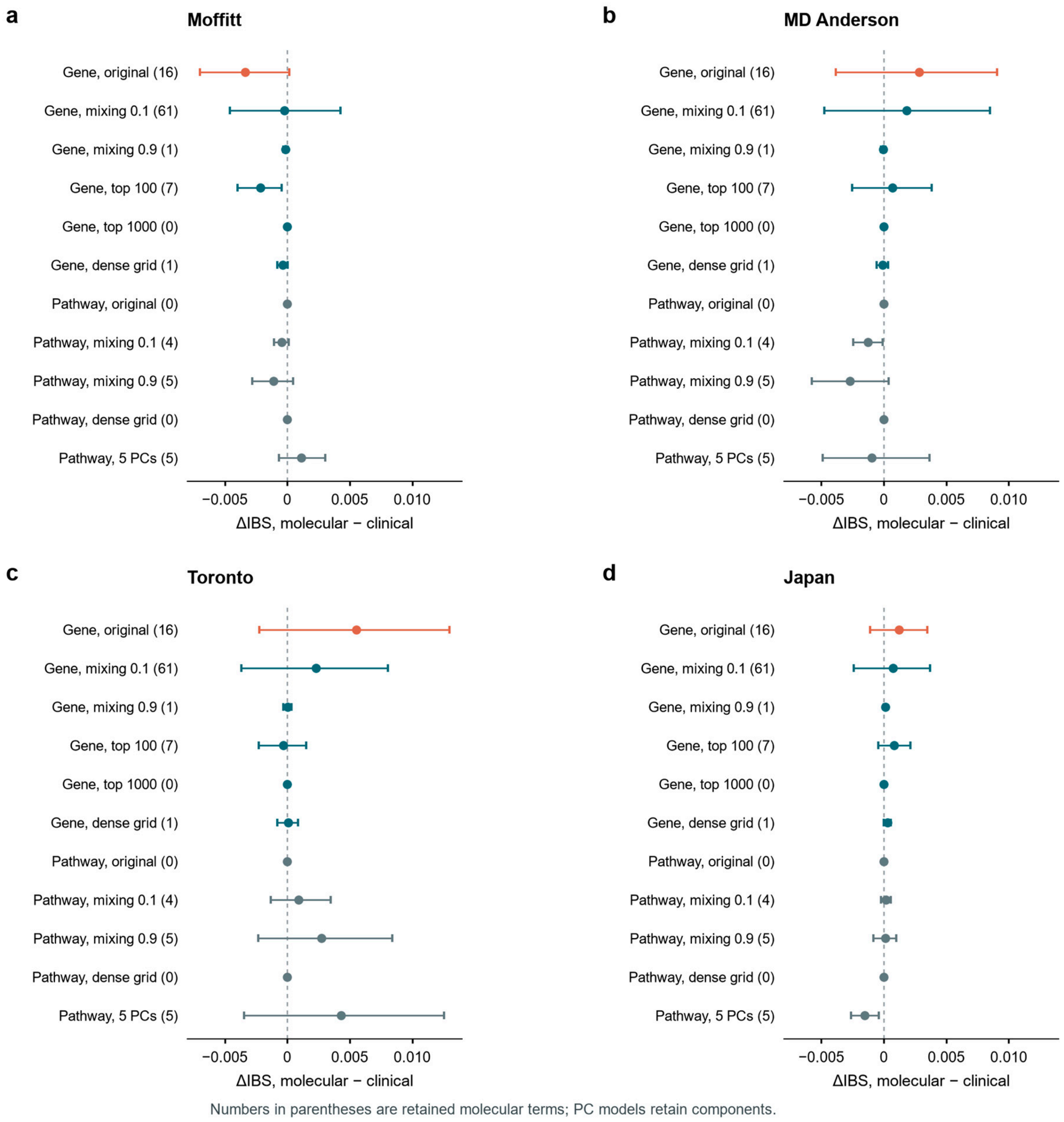

Figure S3. Dependence of external prediction error on modeling specification. Panels show Moffitt, MD Anderson, Toronto and Japan. Points are molecular-minus-clinical IBS contrasts over 1–3 years; bars are multiplicity-unadjusted percentile 95% intervals from 2000 paired within-cohort patient bootstrap resamples with censoring weights re-estimated. Each configuration selected its penalty using development sources only, and intervals condition on the resulting fitted model. The original gene specification is orange, alternative gene specifications are teal and pathway specifications are gray. Numbers in parentheses give the final retained molecular terms; the principal-component model counts components rather than individual pathways. Negative contrasts favor molecular prediction. All 11 assessed configurations are shown; no model was selected from these external results.

Table S30. Prespecified equal-weight summary across the two US evaluation cohorts.

Model	ΔIBS	95% lower	95% upper
Pathway	0.000001	-0.000001	0.000002
Gene	-0.000263	-0.004029	0.003390
Forest	0.001726	-0.004078	0.007948
Boosting	0.002885	-0.003237	0.009392
ISRM_refit	0.000001	-0.000001	0.000002

Moffitt and MD Anderson contribute equal weights. Contrasts are model minus clinical IBS over 1–3 years. Intervals combine independent paired within-cohort bootstrap resamples conditional on the original frozen models.
